# 4D Insights into Coral Biomineralization: Effects of Ocean Acidification on the Early Skeleton Development of a Stony Coral

**DOI:** 10.1002/advs.202508585

**Published:** 2025-09-19

**Authors:** Federica Scucchia, Katrein Sauer, Shah Fara, Tali Mass, Paul Zaslansky

**Affiliations:** ^1^ Department of Marine Biology Leon H. Charney School of Marine Sciences University of Haifa Haifa 3103301 Israel; ^2^ Department for Operative and Preventive Dentistry Charité‐Universitätsmedizin 14197 Berlin Germany; ^3^ Present address: Department of Biological Sciences University of Rhode Island Kingston 02881 United States

**Keywords:** amorphous calcium carbonate, biomineralization, ocean acidification, stony corals, tomography

## Abstract

Coral biomineralization drives the formation of reef structures, but ocean acidification (OA) threatens this process. Coral survival requires effective skeletogenesis in early life stages, through the formation of co‐joined growth zones: rapid accretion deposits (RADs) and thickening deposits (TDs). Contrasting theories and lack of data on how these zones form hamper our understanding of normal coral growth and under future OA. This study describes growth patterns of RADs and TDs during the early stages of coral calcification under both normal and OA conditions. The work reveals geometric characteristics of RADs and TDs at micro‐ and sub‐micrometer scales, as a basis for learning how OA impacts the early‐formed skeletons. By combining material science approaches and Monte‐Carlo simulations to model electron interactions that probe mineral phase composition, we show how TDs and RADs form simultaneously, challenging the classical “step‐by‐step” growth hypothesis. Unexpectedly, under normal pH, TDs comprise ≈65% amorphous calcium carbonate (ACC) and only 35% crystalline aragonite. Under OA, skeletons exhibit higher densities, with only 50% ACC. RADs are underdeveloped under OA, reducing skeletal bending resistance and increasing fracture risk. These findings reveal that the effect of OA on coral skeletogenesis is more complex than previously understood.

## Graphical abstract

Early coral skeletons grow through formation of two co‐joined zones, rapid accretion deposits and thickening deposits. Advanced imaging and simulations reveal that ocean acidification reshapes these zones, shifting the coral portfolio of mineral phases which alters skeletal resistance to mechanical stress. These findings challenge long‐held views of corals growth and uncover key vulnerabilities in reef‐building corals under future ocean conditions.

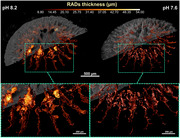

## 1 Introduction

Reef‐building corals are highly sensitive to changes in seawater chemistry, such as those driven by ocean acidification (OA).^[^ ^1^ ^]^ For marine calcifiers such as stony corals, an increase in seawater acidity typically decreases the rate of mineral deposition and yields macroscopically thinner and weaker skeletons.^[^ ^2^, ^3^, ^4^ ^]^ This undermines the robustness of the calcareous structures that must withstand underwater mechanical stresses, a requirement that is fundamental to the survival of entire reefs. Understanding the early stages of skeleton formation is therefore important not only for environmental conservation but also for biomimetics. Detailed knowledge of coral calcification is critical for establishing baselines to assess current and future impacts of the environment, for developing and refining conservation strategies, and for monitoring their effectiveness over time through changes in skeletal growth. At the same time, the intricate architecture details of the skeleton may reveal the efficiency of biomineralization processes while providing inspiration for biomimetic applications. In fact, development of advanced materials may be inspired by coral mineralization, which is of great interest in engineering sciences. To date, little is known about the effects of OA on the micro‐to‐nano length scales of the growing skeletons, and how such changes may directly relate to the structural integrity of stony coral growth. This is especially true regarding possible modifications to the dynamics of the formation of rapid accretion deposits (RADs) and thickening deposits (TDs), the two main growth zones that jointly shape stony coral skeletons.^[^ ^5^, ^6^ ^]^ RADs and TDs begin forming in the earliest coral life‐stages, expanding to form large colonies of multiple polyps. Each polyp builds a calcium carbonate cup around itself, which provides structural support and protection. Coral survival is dependent on sustained skeletogenesis from these earliest life stages, because successful settlement and growth ultimately ensure the prosperity of the entire reef.^[^ ^7^ ^]^

**Figure 1:**
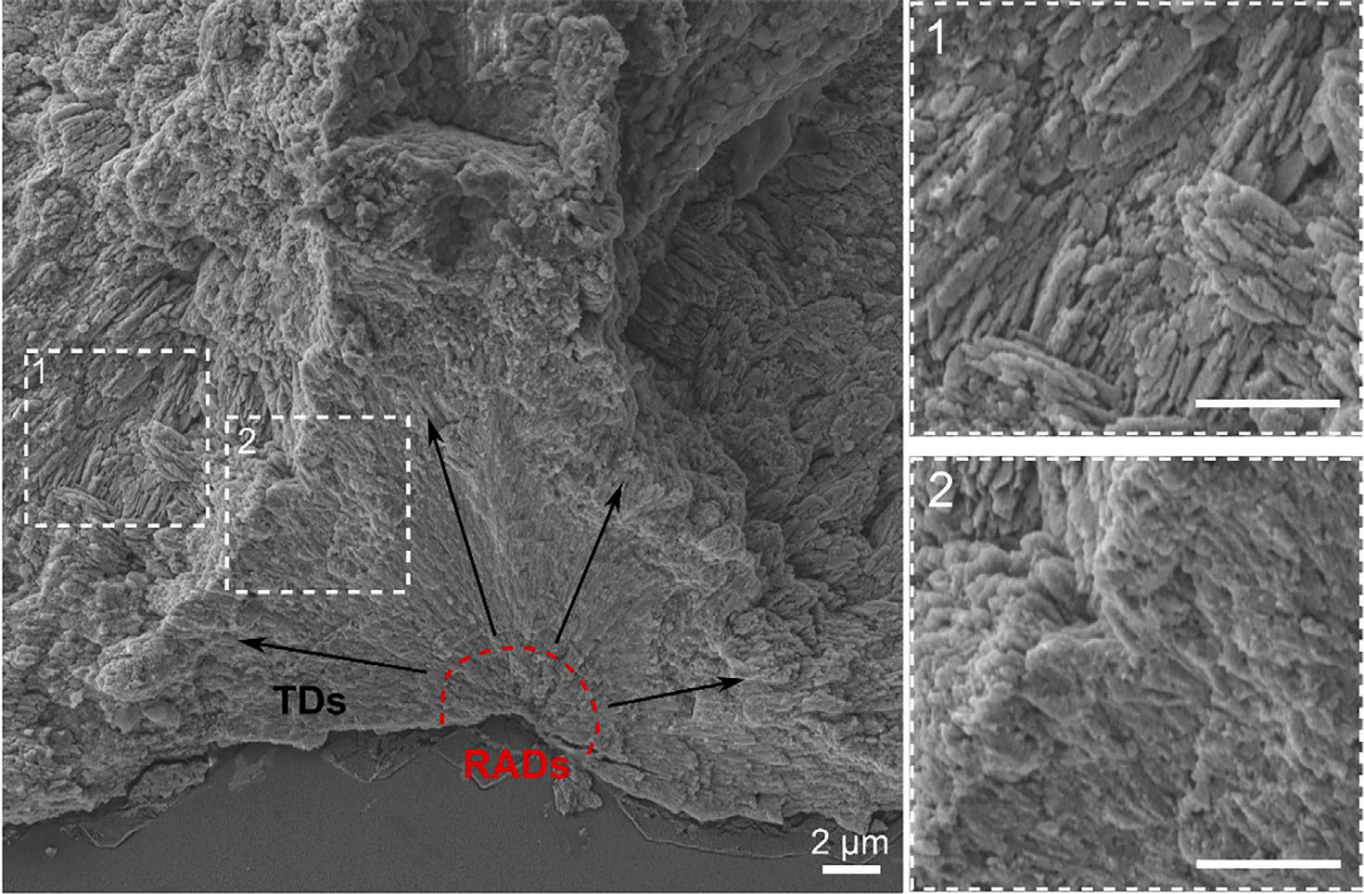
Coral skeletal microstructures: rapid accretion deposits (RADs) and thickening deposits (TDs). Scanning electron microscope (SEM) image of typical fractured cross‐sections of a coral polyp septum, showing the arrangement of TDs radiating outward from central RADs. Insets reveal the TDs fiber arrangement on the skeleton surface (1) and along a fracture surface (2).

RADs, also known as “calcification centers”, are regions of the coral skeleton consisting of disordered granular aggregates of calcium carbonate made of crystalline aragonite as well as other, mainly amorphous calcium carbonate (ACC) phases.^[^ ^8^, ^9^, ^10^, ^11^, ^12^ ^]^ RADs are often considered to be core regions of the coral skeleton where calcification is initiated.^[^ ^9^ ^]^ The bulk of the coral skeleton is made of TDs, also known as “fibers,” consisting of tightly‐packed elongated crystals, primarily aragonite, that characteristically radiate away from RADs^[^ ^8^, ^9^, ^13^, ^14^, ^15^ ^]^ (**Figure** **1**). The literature describes a “step‐by‐step model” of coral skeleton growth, that stipulates that RADs develop before TDs,^[^ ^9^ ^]^ but this concept has been challenged by a “layered model”,^[^ ^6^ ^]^ which suggests that RADs and TDs form simultaneously. Yet so far, there is no consensus on which of the two models correctly describes natural coral skeleton formation dynamics. Such knowledge is essential for making progress in better understanding biological control over biomineralization processes.

Though it is long known that aragonite is the main CaCO_3_ mineral phase of coral, RADs and TDs also contain precursor ACC phases. The presence of ACC has been reported in many CaCO_3_‐forming organisms,^[^ ^8^, ^10^, ^12^, ^16^, ^17^, ^18^, ^19^, ^20^, ^21^ ^]^ and in corals it is assumed to precede or template the growth of aragonite crystals,^[^ ^17^ ^]^ possibly via transient hydrous calcium carbonate phases.^[^ ^12^ ^]^ However, it is not known how ACC is related to the growth dynamics of TDs and RADs in the coral skeleton. Such information is particularly lacking about the earliest and most delicate coral growth stages, that are decisive for future reef growth.

Here, we examine the first‐formed material created in early coral polyp stages to reveal the spatio‐temporal dynamics of RADs and TDs deposition, while quantifying how much of the mineral in these domains is actually crystalline aragonite. We further reveal how and to what extent the skeleton changes in corals reared under acidic pH. We focus on primary polyps, the initial coral calcifying stage, of *Stylophora pistillata*, an ubiquitous Indo‐Pacific stony coral species, grown under ambient and under acidic conditions (pH 8.2 and 7.6, respectively; NBS scale) predicted to occur by the end of the 21st century under the high greenhouse gas emission scenario RCP8.5.^[^ ^22^, ^23^ ^]^ Here, we bring together results from scanning electron microscopy (SEM), synchrotron X‐ray µCT, X‐ray diffraction (XRD), and X‐ray fluorescence (XRF) enhanced with Monte‐Carlo simulations, to understand the behavior of backscattered electrons within coral skeleton material. This made it possible to determine the mineral phase composition, while revealing new sub‐micrometer insights into the 3D mineral growth dynamics. Our results provide insights into newly formed coral skeletons including mineral characteristics, growth patterns and geometric relations of RADs and TDs under normal and acidified oceans.

## 2 Results

### 2.1 RADs and TDs have Different Densities that Vary Under OA

**Figure 2:**
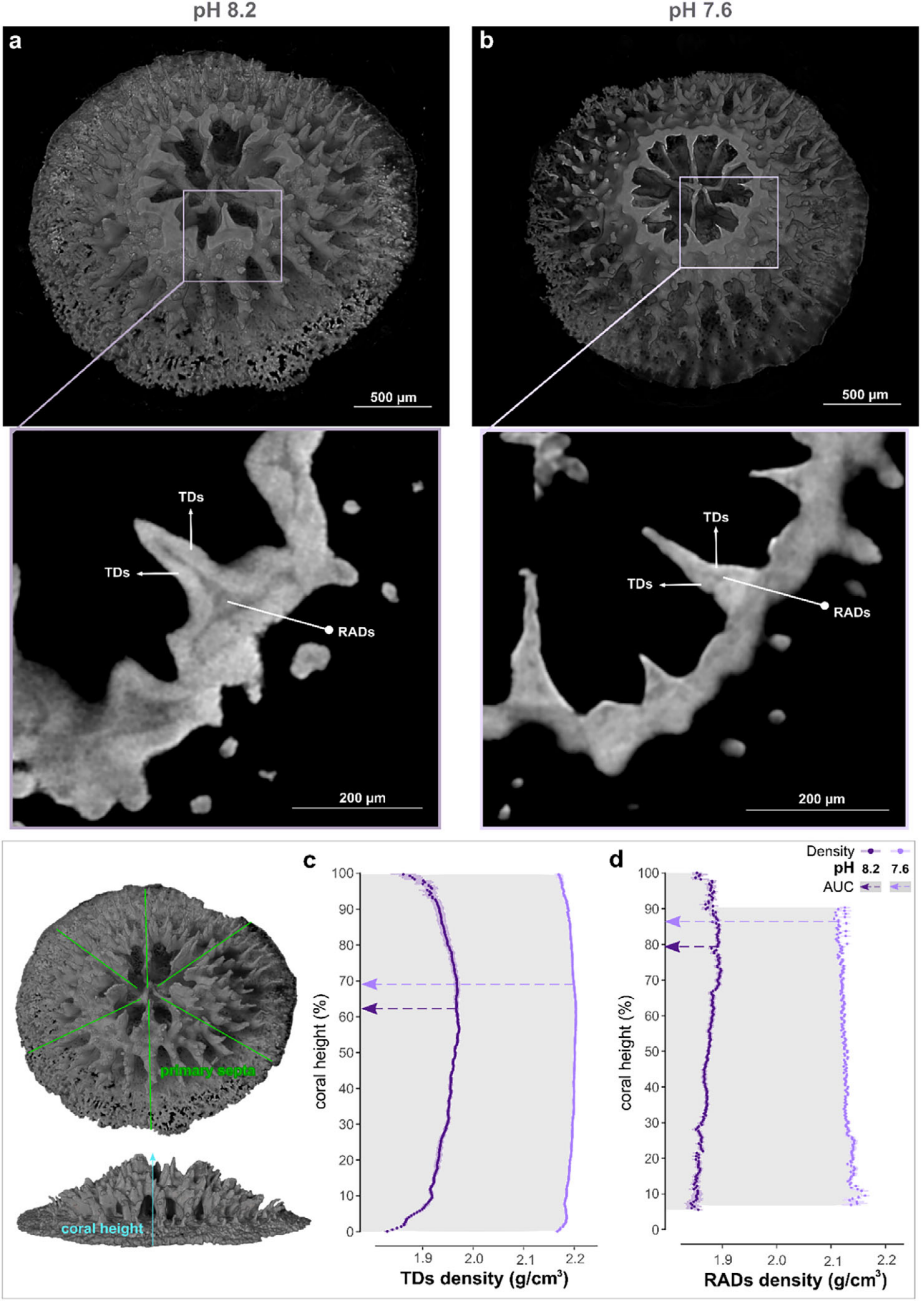
Density variations in entire TDs and RADs across multiple coral primary septa. a,b) Example 3D renderings of entire primary polyp skeletons grown under normal (a) and OA (b) conditions, as well as example transverse planes revealing RADs and TDs (insets) found within each primary septa. c,d) Density distributions along the primary septa (green lines marked on the coral polyp 3D rendering on the left) of TDs (c) and RADs (d) from the skeleton base to the top. Values shown depict means ± standard error of the mean for *n* = 18 septa, *n* = 6 septa per polyp grown under each pH condition. The shaded grey areas to the left of the lines indicate the portions of the coral heights where differences between corals at pH 8.2 (purple arrows) and 7.6 (lilac arrows) are statistically significant, as measured by comparing the area under the curve (AUC)(in these graphs it is the area enclosed within the y axis and the data points; Mann–Whitney test, *p* < 0.05; Table S1, Supporting Information).

We examined high‐resolution X‐ray µCT scans and SEM images of skeletons of *S. pistillata* primary polyps, cultured under either natural or experimental OA conditions. We have previously shown that it is possible to detect contrast differences between TDs and RADs.^[^ ^24^ ^]^ These become visible at submicrometer resolutions and in 3D by synchrotron‐based phase‐contrast enhanced µCT, which shows clearly identifiable differences in density between the two skeletal zones in all parts of the coral polyp skeleton.^[^ ^24^ ^]^ To quantify this, we augmented the 3D data by measuring the mineral density of both RADs and TDs using backscatter SEM measurements, which were calibrated against known densities^[^ ^25^ ^]^ (Figure S1, Supporting Information). The SEM imaged surfaces were of domains that were identified in 3D within monochromatic X‐ray µCT absorption data as well as within phase‐contrast enhanced images of the same samples (**Figure** **2**a,b). Such measurements made it possible to calibrate the density distributions, at micrometer resolution, within entire polyp skeletons in 3D. We thus quantified mineral densities in all the TDs and RADs formed by corals grown under normal and OA conditions (Figure 2). The results show that TDs have higher densities compared to RADs when grown under normal pH condition (3.9% higher density in TDs as compared to RADs; statistical analyses in Table S1, Supporting Information). Furthermore, we observe that both TDs and RADs have higher densities under OA conditions compared to the normal condition (Figure 2c,d; density at pH 7.6 is 13.2% and 13.7% higher in TDs and RADs, respectively; Table S1, Supporting Information).

### 2.2 3D Morphometry Shows that Skeleton Development is Reduced under OA and that RADs Cannot Start Forming Before TDs

**Figure 3:**
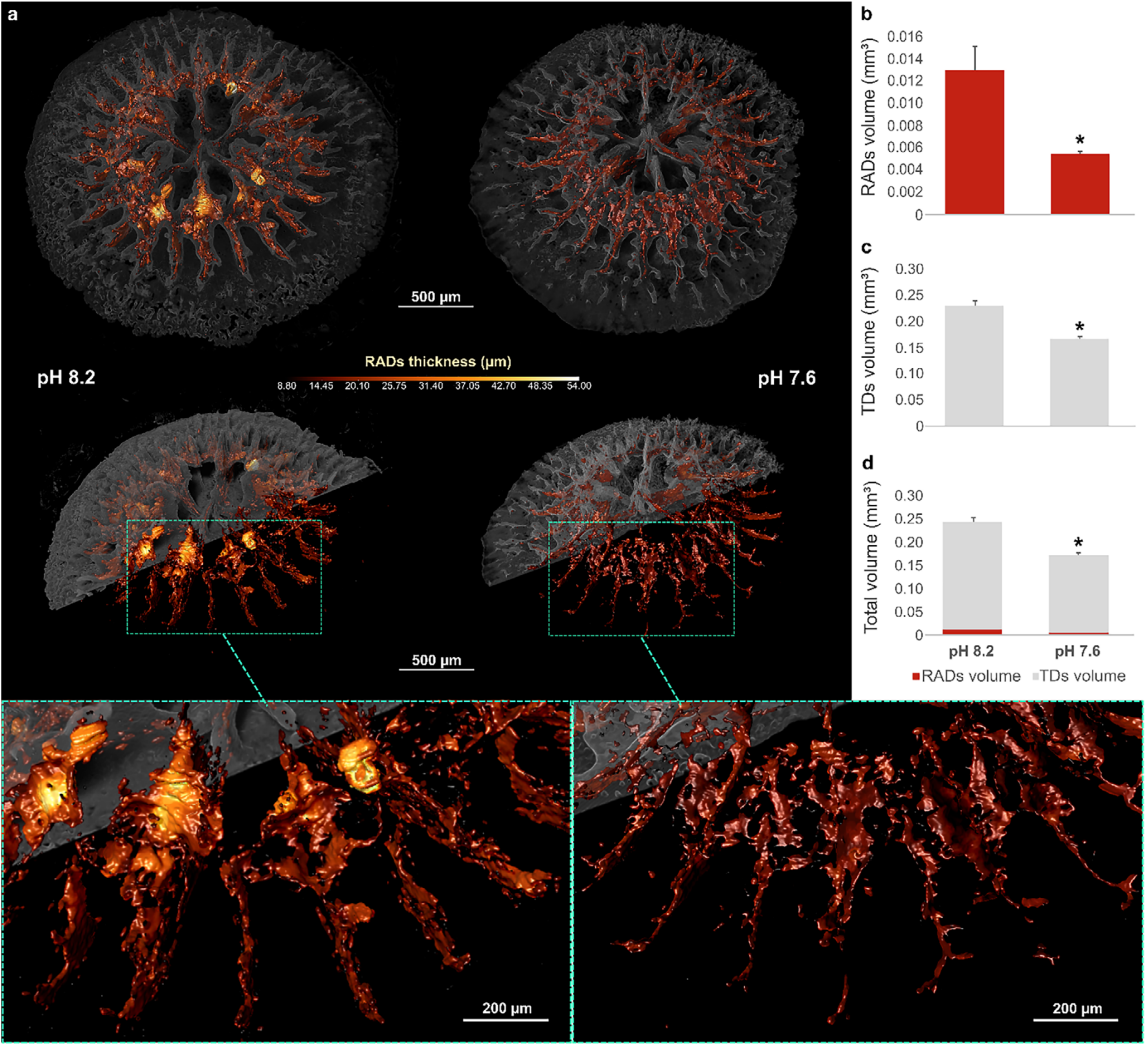
Volumetric arrangement of RADs and TDs within the skeletons of primary polyps. a) Tomographic reconstructions showing the 3D architecture of RADs (red/yellow) and TDs (semi‐transparent gray) in coral primary polyps grown under normal (pH 8.2) and OA conditions (pH 7.6). Magnified views reveal a change in distribution of RADs between the different pH conditions. b,c) Measurements of volumes of RADs and TDs across the entire skeleton d) of coral polyps (values are shown as means ± standard error of the mean, based on *n* = 3.7 × 10^6^ and *n* = 1.6 × 10^6^ voxels per RADs at pH 8.2 and pH 7.6, respectively, and on *n* = 5.8 × 10^7^ and *n* = 4.7 × 10^7^ voxels per TDs at pH 8.2 and pH 7.6, respectively; *n* = 3 polyps per pH conditions were analyzed). Asterisks represent statistically significant differences (Unpaired *t*‐test, *p* < 0.05; Table S1, Supporting Information) between normal and OA conditions.

Phase contrast‐enhanced tomographic reconstructions of the primary polyps clearly reveal an elaborate 3D network of RADs enclosed within the TDs (**Figure** **3**a and Video S1, Supporting Information), which constitute the bulk of the skeleton (Figure 3d). Quantification and inspections in 3D show that under OA conditions, the volumes of both RADs and TDs are significantly reduced (Figure 3a–c), such that the volume of the entire polyp skeleton is decreased (Figure 3d). These 3D observations suggest that exposure to OA strongly affects the development of the skeleton domains in the primary polyps.

**Figure 4:**
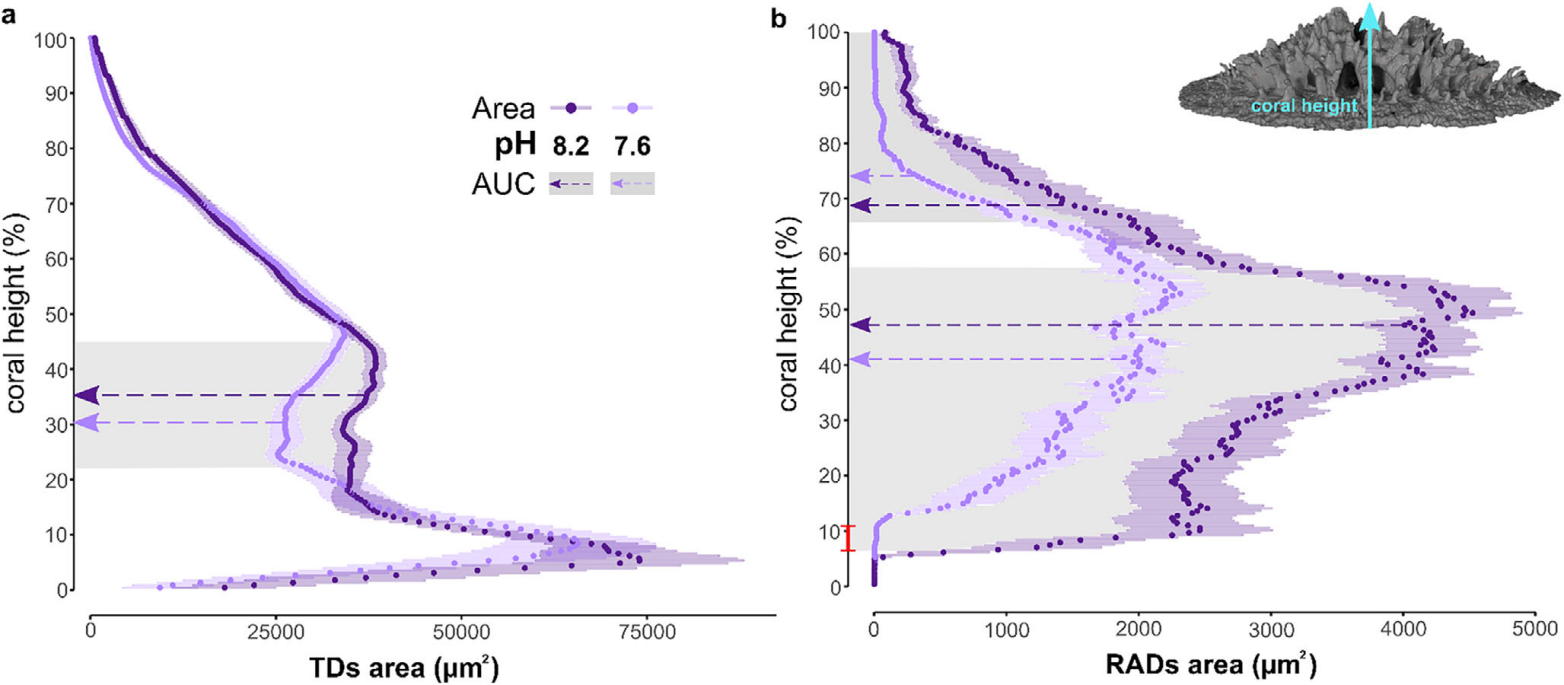
Spatial distribution of RADs and TDs across the primary septa. Cross‐sectional area of a) TDs and b) RADs measured in the primary septa of coral polyps (values are shown as means ± standard error of the mean for *n* = 18 septa, *n* = 6 septa per each polyp at each pH condition). The portion of the coral height (≈5 to ≈11%) highlighted in red in (b) indicates a delay in the initiation of RADs growth under OA conditions. RAD areas in this region are for the most part zero (i.e. totally absent) or near zero in pH 7.6 grown corals. At this height, pH 8.2 grown corals always show well‐developed RADs. The shaded grey areas indicate the portions of the coral height where differences between corals at pH 8.2 (purple arrows) and 7.6 (lilac arrows) are statistically significant, as measured by comparing the area under the curve (AUC)(in these graphs it is the area enclosed within the y axis and the data points; unpaired *t*‐test or Mann–Whitney test, *p* < 0.05; Table S1, Supporting Information).

For a higher resolution evaluation of the morphological characteristics of TDs and RADs, we mapped 3D changes in geometry within the primary septa (**Figure** **4**). These structures are the core supporting columns of the coral skeleton,^[^ ^15^ ^]^ establishing pillars that are present from the base (the zone of attachment of a coral primary polyp to the substrate) to the top of the skeleton. We observe significantly reduced cross‐sectional areas of TDs at pH 7.6 compared to pH 8.2 extending up to the mid‐height of the polyp skeleton (from 20% to 50% of the coral height, Figure 4a). This reduction in the amount of material of TDs corresponds to a decrease at pH 7.6 in both TDs width and length observed at the same 20–50% height region (Figure S2a,c, Supporting Information). When we examine the RADs, we find that at the 20–50% of the septa height region there is an extensive decrease of the cross‐sectional areas at pH 7.6 (Figure 4b). Examining the entire height of the corals, we observed that the cross‐sectional areas (together with widths and lengths) of RADs are significantly reduced in almost all regions of the coral skeletons grown at pH 7.6 (Figure 4b, Figure S2b,d, Supporting Information). This shows that OA has a stronger effect on the RADs development as compared to TDs. Furthermore, it is clear that RADs appear at higher layers (at >10% of the coral height at pH 7.6 compared to ≈5% at pH 8.2; Figure 4b, the region is highlighted in red). Consequently, RADs form later in time during the upward growth of the septa.^[^ ^26^ ^]^ This clearly indicates that RADs formation occurs during or possibly after TDs formation. As a result, RADs cannot serve as seeding regions, as proposed by the “step‐by‐step model” of coral skeleton growth.

### 2.3 Moment of Inertia Decreases and Larger Crystals form Under OA Conditions

**Figure 5:**
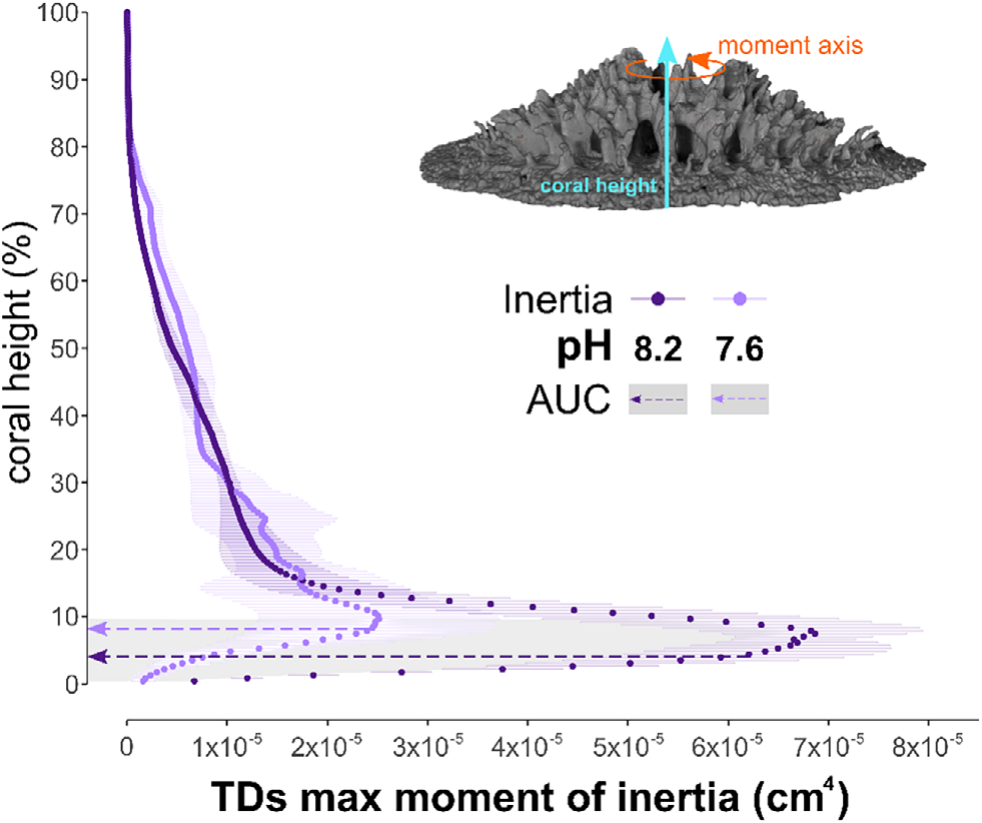
Maximum moment of inertia along the polyp skeleton. Maximum moment of inertia expressed as % of coral height (values are shown as means ± standard error of the mean for *n* = 3 polyps per each pH condition). The shaded grey areas indicate the portions of the coral height where differences between corals at pH 8.2 (purple arrows) and 7.6 (lilac arrows) are statistically significant, as measured by comparing the area under the curve (AUC)(in these graphs it is the area enclosed within the y axis and the data points; unpaired *t*‐test, *p* < 0.05; Table S1, Supporting Information).

Our morphometric 3D observations show that under OA conditions primary polyps form smaller skeletons with different relative distributions of TDs and RADs compared to normal pH condition. We then further explored how such morphological changes may affect coral skeleton mechanical resilience. Using approaches developed for quantification of the mechanical competence of long bones, we examined the maximum moment of inertia, also known as second moment of area, which describes how the object material is distributed around an axis, influencing the resistance of the body to bending.^[^ ^27^ ^]^ A larger moment of inertia indicates that the structure is more resistant to bending under the same externally applied loads. Given that an object will fracture when loaded beyond the bending strength, if we assume coral skeletons all have approximately the same material strength, our maximum moment of inertia analysis provides insights into the capacity of young coral polyps to withstand underwater bending loads without failing. The moment of inertia can be readily computed based on our high‐resolution tomographic data.^[^ ^28^ ^]^ Due to the overwhelmingly larger contribution of the TDs to the total volume of the coral skeleton (Figure 3d), we focused on the maximum moment of inertia calculated based specifically on the TDs data, comparing the geometries created under both pH conditions. We find significantly reduced values in the skeletons grown under OA conditions mainly near the coral base (0 to ≈10% of the coral height, see shaded gray area in **Figure** **5**). Note that this is a measure of how the material is distributed, not the net amount of skeleton produced. The absolute cross‐sectional TDs area near the base of the skeleton is in fact not different between corals grown under different pH conditions (Figure 4a). The reason for this discrepancy is that, under OA conditions, there is an almost complete lack of RADs (Figure 4b), whereas under normal pH conditions RADs readily form (see region highlighted in red, Figure 4b). In other words, coral polyps growing under OA conditions end up having a lower second moment of inertia at the base of the skeleton, and are thus much more likely to break off under bending stresses that they might encounter in the marine environment.

**Figure 6:**
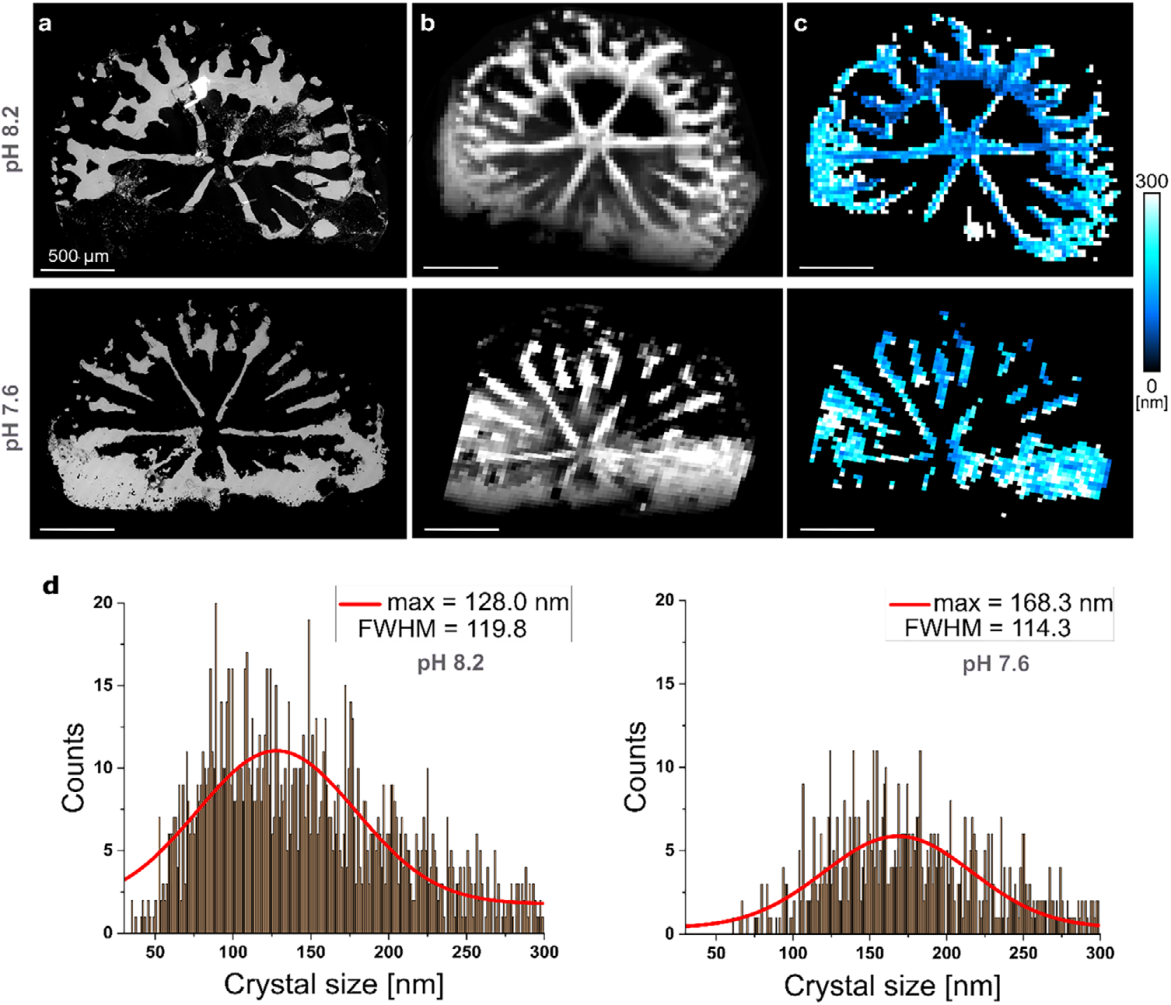
Backscatter SEM, XRF scattering, and crystal size distributions within coral cross‐sections. a) SEM images, b) signal of the total X‐ray fluorescence interactions of the X‐ray beam with the skeleton, and c) XRD‐based crystal size maps of macroscopic cross‐sections of coral primary polyps grown under normal (top) and OA (bottom) pH conditions. d) Histograms of values in (c) illustrate the distribution of crystal sizes for each pH condition. The total number of counts in the crystal size maps corresponds to 1393 and 654 pixels for pH 8.2 and pH 7.6, respectively. FWHM: Full Width Half Maximum.

Our results go beyond observations regarding material distribution and the changes observed in coral resistance to bending, because strength is also influenced by the characteristics of the material that makes up a structure. In nanocrystalline materials, smaller crystals within a given volume of material create more grain boundaries, and these act as barriers to dislocation movement,^[^ ^29^, ^30^ ^]^ which has the effect of increasing the material overall strength.^[^ ^29^ ^]^ This is because the grain boundaries accord higher strength and hardness to polycrystalline solids, generally improving both properties as compared to single crystals. We assessed the characteristics of aragonite CaCO_3_ crystals, specifically the crystal widths distributions, to quantify differences between corals grown under the different pH conditions. **Figure** **6** compares electron microscopy data with 2D microfocus X‐ray maps of the same samples. The latter shows how the embedded and polished coral skeleton material interacts with the incoming X‐ray pencil‐shaped beam, that may be compared with what is observed by SEM. The X‐ray datapoints make it possible to evaluate the characteristic sizes of the crystal unit cells, specifically along the a‐axis (one of the three main directions in the crystal lattice that is commonly used to describe lateral crystal dimensions) of this crystal system. Crystal sizes were thus quantified based on analysis of the aragonite (200) reflections of the nanocrystals (Figure S3; see Experimental Section for additional details on crystal size determination). We observed striking differences between the nanocrystal lateral extent when grown at pH 8.2 and 7.6 (Figure 6c,d). Under acidic conditions, the mean crystal thickness reaches 168 nm as compared with 128 nm, observed in skeletons grown at pH 8.2 (Figure 6d). This ≈30% increase provides a possible material‐based explanation for the density differences that we observed (Figure 2). Larger crystals contain fewer interfaces whereas the smaller crystals provide larger surfaces and more interfaces e.g., with other components that may include remnant amorphous or other calcium carbonate polymorphs that may be present (e.g., calcium carbonate hemihydrate CCHH^[^ ^12^ ^]^). This is therefore an important structural difference between the skeleton material produced by corals grown under normal versus OA pH conditions.

### 2.4 Quantification of ACC Abundance Within the Skeleton

The different densities and crystal sizes of aragonite raise the possibility that there are differences in the amount of crystalline and non‐crystalline CaCO_3_ phases. In fact, small amounts of CaCO_3_ polymorphs other than aragonite have been demonstrated within the skeletons of both adult^[^ ^12^, ^31^, ^32^, ^33^, ^34^ ^]^ and young^[^ ^26^, ^35^, ^36^ ^]^ corals. We searched our X‐ray data for hints of the presence of phases other than aragonite, such as the recently described CCHH and monohydrocalcite (MHC),^[^ ^12^ ^]^ however, the low resolution of our XRD beam and the overwhelmingly strong signal from aragonite revealed only negligible (non‐conclusive) presence. Hence, for the following analysis, we lump all other non‐aragonite contributions to the density into one, second phase, for which we adopt the previously‐used loose term ACC (though not all components are amorphous). Indeed, the predominant mineral phase present in all our primary polyps is aragonite with traces of calcite (Figure S3, Supporting Information). We performed Monte‐Carlo simulations to model backscattered electron interaction within the TDs (Figure S4a, Supporting Information) assuming a mixture of two phases. The backscatter intensity in our experimental electron microscopy measurements correlates with composition and density that electrons “feel” along the propagation path. Therefore, we created different simulations for aragonite in the form of elongated nanocrystals, encased with layers of ACC (which may in fact also be CCHH or other intermediates). Simulations of the different geometries of layers were based on SEM observations of the arrangements of crystals in TDs that show packets of fiber bundles (Figure 1, insets), that are aligned either along or orthogonal to the incoming electron beam (Figure S4b, Supporting Information). We simulated multiple‐layered arrangements of crystalline and amorphous CaCO_3_, to match penetration depths consistent with the SEM experiments (Figure S5, Supporting Information). We make the assumption that, due to a loss of electrons inside the material, the measured intensity is more‐or‐less inversely related to the electron penetration depths. Strikingly, the backscatter intensity measured in the SEM (**Table** **1**) was far lower than expected for pure aragonite. Indeed, electron penetration depths observed in the Monte Carlo simulations show that the skeleton material cannot be made only of aragonite crystals (Table 1). We therefore iteratively changed the model to incorporate increasing thicknesses of ACC (Figure S4b, Supporting Information), for which we assume the reported density of 1.6 g cm^−3^,^[^ ^37^ ^]^ interspersed with aragonite crystals. We found that in corals grown at pH 8.2, a mineralized structure comprising 35% of aragonite and 65% of ACC is required to achieve consistency with the observed SEM measurements of the TDs (Table 1). Interestingly, for corals grown under pH 7.6, the model predicts a structure with 50% aragonite nanocrystals and 50% ACC. These results corroborate our hypothesis that the observed density differences are due to changes in the abundance of CaCO_3_ phases present in the mineral material produced by the corals grown under different pH conditions. These findings show that much of the coral mass created under normal pH is not crystalline aragonite. For completeness, we consider the possible effects of TDs fiber orientation and other possible interactions with electron penetration depths. The orientation of the >1 µm elongated aragonite crystals relative to the incoming electron beam does indeed have a small influence on the simulation results. However, taking into consideration all possible configurations of crystals with respect to the incoming electron beam (Figure S6, Supporting Information), we find that, in all cases, a significant amount of mineral that is not aragonite is required to explain the lower electron intensities that we observed experimentally. As an adjunct, we found that strontium identified in the corals through XRF, was more abundant under normal pH compared to OA conditions (Figure S7, Supporting Information). As strontium cannot easily become incorporated in aragonite crystals, it is assumed to act to stabilize ACC.^[^ ^38^ ^]^ The widespread distribution of this element in our measurements and previous reports finding CaCO_3_ that is not aragonite,^[^ ^12^ ^]^ corroborate our simulations and lead to the conclusion that coral mineral grown under normal pH conditions must contain less aragonite than the skeletons grown under OA conditions.

**Table 1:** Summary of experimental inputs and results for Monte Carlo simulations of the TDs. Crystal sizes calculated from the width of the (200) reflection of aragonite phase (see Experimental Section; values shown as maximum of the Gaussian function overlayed with the histograms), density values quantified by backscatter SEM, and penetration depth derived from Monte‐Carlo simulations are presented for TDs in corals grown at pH 8.2 and 7.6. Simulation results are based on literature reports of density of 2.93 and 1.62 g cm^−3^ for 100% aragonite and 100% ACC, respectively (indicated by asterisks).

TDs		Density [g cm^−3^]	Monte‐Carlo simulations: Backscatter electron penetration depth [nm]
**pH 8.2** crystal size: 128 nm	Backscatter SEM	2.18 ± 0.04	717 ± 5
	Predicted 35% aragonite and 65% ACC	2.93 & 1.62*	712 ± 8
**pH 7.6** crystal size: 168 nm	Backscatter SEM	2.38 ± 0.01	651 ± 4
	Predicted 50% aragonite and 50% ACC	2.93 & 1.62*	654 ± 7

## 3 Discussion

While there is ample evidence for the presence of phases other than aragonite within the coral mineralized material,^[^ ^8^, ^10^, ^12^, ^16^, ^17^ ^]^ it has so far not been considered as an important factor in relation to the geometry and physical properties of the skeleton. The lack of information regarding how much ACC is present in the skeleton has limited our understanding and our capacity to predict coral growth dynamics in more acidic seawater. Our data provides evidence and quantification for the prevalence and importance of varying ratios between aragonite and other, likely non‐crystalline CaCO_3_ phases, within the newly forming coral skeletons. We find that much of the mineral (more than 50% of TDs, that comprise the majority of the skeleton, Figure 3d) is in fact not crystalline aragonite, at least in the early coral polyp growth stages (Table 1). Under OA conditions, there is a shift in the relative abundance of CaCO_3_ phases with a clear increase in the amount of detectable aragonite signal (Table 1). This shift toward more crystalline aragonite and less amorphous phases fully matches the increased density of the skeletal structures (Figure 2). It is important to note that our measurements capture local mineral density at the microscale, based on the relative abundance of crystalline versus amorphous calcium carbonate phases. This is distinct from bulk skeletal density measurements,^[^ ^2^, ^4^, ^39^, ^40^ ^]^ which reflect whole‐skeleton properties including pores, organic content, and macrostructural features. Our data provides the first complementary, high‐resolution insights into mineral phase composition, rather than organism‐scale skeletal mass. Furthermore, our results offer new insights into the skeleton growth of early coral life stages under OA, suggesting that as seawater acidifies, there is a shift in the coral portfolio of mineral phases.

The density increase we observed can also be explained by the deposition of larger crystals (Figure 6). This observation is consistent with general principles of crystallization, where crystal size is determined by the balance between nucleation and growth.^[^ ^41^ ^]^ Under lower supersaturation conditions, such as those potentially occurring under OA, nucleation is less frequent and more energy‐intensive,^[^ ^42^ ^]^ resulting in fewer crystals that can grow larger.^[^ ^43^, ^44^ ^]^ In contrast, higher supersaturation favors rapid nucleation and the formation of many small crystallites.^[^ ^43^, ^44^ ^]^ Thus, the formation of larger aragonite crystals in OA‐exposed corals may reflect a shift toward growth‐dominated crystallization dynamics. Although we did not directly measure the extent or dynamics of ACC formation, it is possible that a potential reduction in the incorporation of ACC under OA conditions may have also contributed to the observed increase in skeletal density. This is unlikely to be due to ACC dissolution, as we did not observe any evidence of physico‐chemical dissolution of the polyp skeletons. This aligns with previous experiments that exposed live corals to even lower pH levels (as low as pH 2–4) without observing signs of dissolution,^[^ ^2^ ^]^ and it is in agreement with the critical role of the living coral tissue that protects the underlying skeleton from acidic seawater.^[^ ^45^ ^]^ Thus, as long as pH and the aragonite saturation state at the site of calcification are controlled by the coral,^[^ ^46^, ^47^ ^]^ the suite of skeletal modifications we observed in the skeleton under OA are more likely to reflect a biologically mediated calcification response, rather than passive dissolution of pre‐existing skeletal material.

An additional factor to consider is the role of the skeletal organic matrix, traces of which become entrapped within both RADs and TDs.^[^ ^5^, ^6^, ^8^, ^9^, ^11^, ^13^, ^48^ ^]^ While we did not carry out analyses to quantify changes in the organic matrix content, we acknowledge that there may be a potential influence on mineralization. Nonetheless, organic material constitutes a relatively small fraction of the total coral skeletal mass, typically 0.8–1% and up to 2.5% when including associated water.^[^ ^49^ ^]^ Furthermore, changes in organic matrix content under OA have been shown to correspond to only ≈3.56 µg cm^−3^,^[^ ^2^ ^]^ which is orders of magnitude smaller than the mineral density differences observed here (0.2–0.3 g cm^−3^). Therefore, it is unlikely that changes in organic content alone can explain the density differences that we found between normal pH and OA growth conditions. Building on this, we next consider how the observed changes in the density and aragonite content relate to the other differences that we observed in the RADs and TDs growth, in particular the reduced moment of inertia of TDs found under OA conditions (Figure 5). One of the causes of this reduction can be tracked back to the scarce presence of RADs under OA conditions (Figure 4b), thus we conclude that having less RADs may undermine structural resilience of the skeleton. This makes it possible to try to answer the long‐standing question “why do corals have RADs” and specifically, we may now know what their evolutionary role could be. Various researchers have considered RADs to be the seeding regions for TDs,^[^ ^8^, ^9^, ^13^, ^14^, ^15^ ^]^ but as we show here, TDs are also found in regions where RADs have not formed, which becomes even clearer in corals grown under OA conditions (Figure 4). This finding supports the “layered model” of coral skeleton growth.^[^ ^6^ ^]^ So, if RADs are not the source of growth for TDs, why do corals form both RADs and TDs? Our bending resistance data provides clues that may answer this question, and our results indicate that the primary role of RADs within the coral skeleton is likely linked to the need for rapid enhancement of skeletal mechanical resistance to bending, so as to acquire the geometry needed to reach sufficient strength. This might be similar to the role of layers that are present in mollusks shells, which are indispensable to improve the whole strength of the shell.^[^ ^50^, ^51^ ^]^ In those organisms, the region of the multi‐layered shell where the inner layer is missing is the most vulnerable part of the whole structure.^[^ ^50^ ^]^ As future work, it would be important to investigate mechanical properties of the coral skeleton based on the presence and size of RADs within other coral species and developmental stages, paired with finite element modeling for computational mechanical testing, to address what stresses develop depending on microstructure characteristics. We note that we still do not know how OA may affect the biological and genetic control over the RADs formation, though it is known that the growth of these regions is controlled, in part, by acid‐rich proteins of the CARP family^[^ ^11^ ^]^ and that the expression of the genes encoding these proteins is sensitive to pH changes.^[^ ^52^, ^53^ ^]^

Even though in primary polyps the uptake of seawater from the external environment is rapid,^[^ ^54^ ^]^ corals have control over the chemistry of their calcifying space,^[^ ^46^, ^47^ ^]^ and can in some cases buffer decreases in seawater pH.^[^ ^55^, ^56^, ^57^, ^58^ ^]^ However, as ocean acidity keeps increasing, the concentration of protons in seawater also rises, steepening the proton concentration gradient between the coral calcification site and the surrounding seawater. Thus, the energy required to transport protons across a larger gradient is expected to increase, making the coral skeleton deposition more energetically demanding.^[^ ^59^ ^]^ As an organism tolerance to stress is energy‐limited,^[^ ^60^ ^]^ there will be a point when it becomes difficult for the coral to tightly control the chemistry of the calcification site, therefore slowing down the skeleton growth. This energetic mismatch may be the underlying cause leading to the formation of larger crystals and smaller mineralized structures (Figures 3 and 4), and to corals not being able to reach the same size within the same amount of growth time as is seen under normal pH conditions (Figure 3). A weaker skeleton and slower growth is likely to render young corals more susceptible to predation and competition, jeopardizing their fitness.^[^ ^61^ ^]^ However, further assessments are needed to evaluate if the skeletal changes observed here are maintained through adulthood and are shared across species with different growth forms. Undoubtedly, stony corals will be facing multiple threats in the coming decades due to eutrophication, thermal stress, and seawater acidification.^[^ ^62^ ^]^ Yet, these organisms have experienced and survived major environmental changes throughout geologic time, including extensive fluctuation in atmospheric CO_2_ levels, temperature, and seawater pH conditions.^[^ ^63^, ^64^ ^]^ In this context, the mineral phase plasticity that we observe in early calcification may represent one potential mechanism contributing to coral resilience. While it remains unclear how broadly such responses can buffer corals against future change, our results suggest that some coral species retain a degree of structural flexibility that may be part of a broader suite of responses available to the organism to ensure survival under changing conditions, at least during early developmental stages. Thus, lessons across geological time scale and from our observations suggest that there is potential for corals to persist under future changing climate, although adaptation to changing ocean conditions will likely drive a shift in the species as we know them today.

## 4 Experimental Section

### Sample Collection and Ocean Acidification Experiment

Coral larvae were collected from adult colonies of the stony coral *S. pistillata* on the reef adjacent to the Interuniversity Institute of Marine Sciences (IUI, 29°30′06.0″ N 34°54′58.3″ E) in the Gulf of Eilat (Israel). Larvae were collected using dedicated larval traps from 15 randomly selected colonies from the shallow reef (depth 6–7 m) for several nights of spawning during April 2020. All live larvae were pooled together and transported to a controlled environment aquarium system at the Leon H. Charney School of Marine Science (University of Haifa, Haifa, Israel). Larvae were put in custom‐made polypropylene plastic chambers (≈20 larvae per chamber) which were placed in a system of six flow‐through aquariums with artificial seawater (1 chamber per aquarium) replicating the spring northern Red Sea water conditions, recorded monthly by the Israel National Monitoring Program of the Gulf of Eilat and previously reported.^[^ ^24^ ^]^ Before inserting the settlement chambers, the carbonate chemistry of seawater was manipulated in three of the experimental aquariums by injecting CO_2_ to reduce the ambient pH 8.2 (pCO_2_ ≈ 487 µatm) and obtain the target value of pH 7.6 (pCO_2_ ≈ 1938 µatm). This target pH was chosen to simulate the global mean surface‐ocean decline in pH predicted to occur by the end of this century under the high greenhouse gas emission scenario RCP8.5, considering that in subtropic oceans the decline was predicted to be higher than other regions.^[^ ^22^, ^23^ ^]^ Water conditions within the aquariums were monitored daily throughout the experiment as previously described.^[^ ^24^ ^]^ Parameters of seawater carbonate system were calculated from pH, TA, salinity, and temperature using the CO2SYS package^[^ ^65^ ^]^ with constants from ref. [66] as refit by ref. [67] (Table S2, Supporting Information). The experiment lasted for a total of 9 days, following the previous experimental procedure.^[^ ^24^ ^]^ The experiment was run for long enough to a) inspect coral recruits with sufficient deposited skeleton to be able to detect changes at macro‐ and micro‐scale levels,^[^ ^26^, ^68^ ^]^ b) analyze coral recruits still at the primary polyp stage, before the start of asexual formation of new polyps. At the end of the experiment, primary polyps were gently removed from the chambers and stored with 90% ethanol in 2 mL tubes for morphological analyses.

### X‐Ray µCT Imaging

Primary polyps were fixed on top of polypropylene micropipette tips with EpoFix resin (Agar Scientific, Stansted, UK). Microtomography scanning of the skeleton material (*n* = 3 per pH treatment, 1 polyp from each tank) was conducted at BAMline,^[^ ^69^ ^]^ the imaging beamline at the synchrotron electron storage ring BESSY II operated by the Helmholtz‐Zentrum Berlin für Materialien und Energie (HZB), in Berlin, Germany. Each sample was attached to a metal stub and scanned with incremental rotation using a high‐resolution imaging setup^[^ ^70^ ^]^ with exposure times set to 1 s per angle, and sample‐detector distance of up to 60 mm, producing multiple projections spanning 180° (step angle of 0.25°) with a final pixel size of 2.2 µm. For each sample, multiple datasets were acquired, each with ca. ≈1000 projections. The phase contrast‐enhanced microCT data was obtained at an energy of 24.5 keV to highlight edges and internal boundaries within the samples, whereas absorption imaging mode at an energy of 15.3 keV, data used to quantify variations in mineral density.

Treatment of the BAMline data including normalization and reconstruction followed the procedure described in ref. [24]. Briefly, each radiograph was normalized to account for beam inhomogeneities using an in‐house pipeline for background‐correcting radiograms with best‐fitting (flat‐field) images, after subtracting dark‐current images (i.e., detector noise captured with the shutter closed with no X‐ray exposure). Reconstruction was carried out with the filtered back projection method using nRecon (v1.7.4.2, Brucker micro‐CT, Kontich, Belgium) to produce 3D datasets from cross‐sectional 2D tomographic images. In nRecon, the best misalignment compensation value was computed for each dataset, together with the best value for ring artifacts removal. Tomographic reconstructed datasets were further processed in 3D using the commercially available software Dragonfly (v2021.3, Object Research Systems‐ORS, Montreal, Canada),^[^ ^71^ ^]^ with further stack analyses performed using FIJI.^[^ ^72^ ^]^

### Scanning Electron Microscope Density Calibration

Primary polyps that were imaged with X‐ray µCT were subsequently polished using a 0.05 µm diamond paste. The polished skeleton was imaged together with an enamel sample using a scanning electron microscope (SEM, PhenomXL, ThermoFisher, Eindhoven, Netherlands) by backscatter imaging at 15 and 20 kV in low vacuum mode, employing a working distance of 15 mm. Density of TDs and RADs within the SEM images were measured using FIJI,^[^ ^72^ ^]^ by delimiting and computing the gray value mode of enamel regions and of TDs and RADs regions and using the following equation:

(1) $$\left[\left(T\;\mathrm{or}\;R-B\right)/\left(E-B\right)\right]*2.8\;$$

where T is TDs, R is RADs, B is background, E is enamel and 2.8 is the density of a small slab of bovine enamel, determined by direct measurements of volume and weight. The same regions measured in the SEM were identified and measured in the X‐ray absorption datasets (Figure S1a–h, Supporting Information). Gray values (mode per each area measured) from the absorption images and density values from SEM images were used to calibrate density for both TDs and RADs at the normal and acidic pH conditions (Figure S1i–l, Supporting Information). Calibration values were used to quantify the density of TDs and RADs within all primary septa, by a) masking the absorption data using the extracted ROI (detailed below) and b) delimiting each septa and computing the gray values using the ROI Multi Measure function within FIJI.^[^ ^72^ ^]^

### Tomographic Image Analyses

Identification of TDs and RADs in tomographic datasets was possible using phase‐contrast enhancement, which delineates the edges and internal boundaries of structures with different densities within the skeleton through strong differences in contrasts.^[^ ^24^ ^]^ When clear boundaries were present between different features in the tomographic dataset, image segmentation can be applied to allow for volumetric quantification and 3D visualization of TDs and RADs, using highly sensitive deep learning‐based approaches.^[^ ^24^ ^]^ Based on this, TDs and RADs were identified in the phase contrast‐enhanced tomography data using an Artificial Intelligence (AI)‐based approach. The Deep Learning functions built into Dragonfly (v2021.3, Object Research Systems‐ORS, Montreal, Canada)^[^ ^71^ ^]^ were employed to objectively classify (segment) and locate TDs and RADs across tomographic datasets, as detailed in ref. [24]. Based on the segmented regions of interest (ROIs), Dragonfly was employed to estimate volumes of TDs and RADs within the skeleton of the primary polyps.

ROIs comprising TDs and RADs were used to mask and separately extract each region from the tomography datasets (both from phase contrast‐enhanced and absorption data). The extracted ROIs were used to compute area, width, and length of TDs and RADs within the primary septa using FIJI,^[^ ^72^ ^]^ by delimiting each septa (*n* = 6 per polyp) in the phase contrast‐enhanced tomography data and applying the ROI Multi Measure function. Width and length were measured as the primary and secondary axis of the best‐fitting ellipse circumscribing TDs and RADs within each tomographic slice. Lastly, extracted ROIs were used to measure the maximum moment of inertia using the Bone Analysis module^[^ ^28^ ^]^ of Dragonfly.^[^ ^71^ ^]^

### Sensitivity of AI‐Based Segmentation

SEM images were also used to assess the sensitivity of the AI‐based segmentation, specifically to estimate the precision of the TDs and RADs identification within the tomographic images. To this end, the area of the same arbitrarily‐chosen regions within TDs and RADs (n = 12 per pH condition) was measured in the segmented data and in the SEM images (Figure S8a–h, Supporting Information) using FIJI.^[^ ^72^ ^]^ For the control pH, the mean area difference (in percentage) between the segmented‐derived regions and the SEM‐derived regions varies between 0.69% for TDs and 0.70% for RADs (Figure S8i, Supporting Information). For the acidic pH, the mean area difference varies between 0.72% for TDs and 0.50% for RADs (Figure S8j, Supporting Information).

### X‐Ray Diffraction and Fluorescence Analyses

For mineral crystal‐phase measurement and crystal size determination, and to resolve mineral composition patterns, embedded thin‐polished coral samples were analyzed using synchrotron‐based fluorescence (XRF) and diffraction (XRD) at the mySpot beamline of the BESSY II synchrotron light source (HZB‐ Helmholtz‐Zentrum, Berlin, Germany) (Figure S9, Supporting Information). Samples were mounted on a sample stage (Figure S9, Supporting Information) and scanned in air using X‐Ray beam energy of 18 keV, a sample‐to‐detector distance of ≈34.4 cm, and a beam size of ≈25 µm. Diffraction patterns were collected using a 3269 × 3110 pixels Dectris M9 Eiger detector with a lateral pixel size of 75 µm. Fluorescence patterns were collected using a RAYSPEC Sirius SDD detector (8 µm beryllium window). The detector orientation, rotation, and sample‐to‐detector distance were calibrated using powdered corundum (Al_2_O_3_) as standard.

Diffraction pattern analysis was conducted with the XRDUA software package v764,^[^ ^73^ ^]^ which included the refinement of the detector calibration parameters (sample‐to‐detector distance, beam center, and tilt angle). Diffraction patterns of well‐defined aragonite and calcite phases (Figure S9, Supporting Information) enable the extraction of peak intensity maxima from the (200) reflections. The a‐lattice parameter of aragonite and crystal sizes were determined via line profile analysis using Voigt fitting^[^ ^74^, ^75^ ^]^ with peaks assigned as previously reported.^[^ ^76^ ^]^ The crystal size distributions derived from (200) peaks were analyzed using OriginPro software (2023, v10.0.0.154, Academic) and converted into 2D maps with FIJI. Histograms of crystal size distributions across the sample cross‐section were fitted with Gaussian functions to determine the most probable crystal sizes for corals grown at pH 8.2 and 7.6. The (111) reflections were used to generate 2D maps of crystal phase distributions. For 2D mapping of XRD and XRF, in‐house FIJI macros were employed to visualize elemental distribution (XRF) and the established mineral phases (XRD) of aragonite with traces of calcite (XRD). As standard practice, intensities in the maps were corrected by subtracting background intensity, which was not related to the detected peak.

### Amorphous and Crystalline Phases Content Determination

Monte Carlo simulations of electron trajectories in models of the coral, comprising for simplicity only CaCO_3_, were performed using the CASINO modeling tool (v2.51).^[^ ^77^ ^]^ Simulations were conducted using electron energies of 20 kV, tracing 50 000 electron trajectories within each simulated substrate, using a beam radius of 25 nm. A substrate thickness of ≈5000 nm was used and different simulations employed that included varying compositions of aragonite (100%, 90%, and 50%), arranged in horizontal and vertical orientations of long and slender nanocrystals (Figure S4b, Supporting Information), with a thickness as determined by the XRD line analysis. For the crystalline and amorphous phases, densities of ρ = 2.93 g cm^−3^,^[^ ^78^, ^79^ ^]^ and ρ = 1.62 g cm^−3^,^[^ ^37^ ^]^ were used and the model (simulated layering of crystals within ACC) electron depth was adapted to match SEM experimental measurements (Table 1 and Figure S5, Supporting Information).

Monte‐Carlo simulations and the experimental determination of TDs densities were based exclusively on backscattered electrons. Electrons that remain within the sample were excluded from the analysis. The trajectories of backscattered electrons, influenced by the atomic number, crystal structure, orientation, and surface texture of the material, can result in variable exit angles. Generally, higher atomic numbers lead to greater electron deflection toward the detector. However, the Monte‐Carlo program used (CASINO) does not account for variations in exit angles (Figure S6, Supporting Information), simplifying and allowing us the comparison of electron penetration depths across different materials.

### Statistical Tests

Statistical significance of the differences between the normal and the acidic pH conditions were assessed by a) computing the Area Under the Curve (AUC) per each septa area, septa width, septa length, septa density (for the density, TDs at pH 8.2 vs TDs at pH 7.6, and RADs at pH 8.2 vs RADs at pH 7.6), for the maximum moment of inertia and b) applying an unpaired *t*‐test or Mann–Whitney test (if the assumptions of normality, Shapiro–Wilk test, and homogeneity of variance, Levene's test, were not met) between the computed AUCs. For the TDs vs RADs density comparison at the normal pH 8.2, all septa density measurements were pooled together per each region and the difference was tested for statistical significance using a Mann–Whitney test (not normally distributed). All statistical test results are reported in Table S1 (Supporting Information). The GraphPad Prism software v8.0.2 (GraphPad Inc.) and RStudio (v2024.04.1) were used to perform statistical tests.

## Conflict of Interest

The authors declare no conflict of interest.

## Author Contributions

T.M. and P.Z. contributed equally to this work. F.S., T.M., and P.Z. conceived the idea for this study. F.S., K.S., S.F., T.M., and P.Z. designed the methodology. F.S. collected the coral samples, conducted the acidification experiment, and analyzed the tomography data. K.S. analyzed the XRD and XRF data and performed the Monte Carlo simulations. S.F. analyzed the SEM data. F.S. led the writing of the paper. All authors contributed critically to the drafts and gave final approval for publication.

## Supporting information



Supporting Information

Supplemental Video 1

## Data Availability

The data that support the findings of this study are openly available in Zenodo at https://zenodo.org/records/10926898, https://zenodo.org/records/14957066, https://zenodo.org/records/14958455, reference number [53].
