# Supplementary material for: 4D Insights into Coral Biomineralization: Effects of Ocean Acidification on the Early Skeleton Development of a Stony Coral
Source: Adv Sci (Weinh). 2025 Sep 19;12(48):e08585. doi: 10.1002/advs.202508585 (PMC12752662; doi:10.1002/advs.202508585)

Supporting Information for

**4D Insights into Coral Biomineralization: Effects of Ocean Acidification on the Early Skeleton Development of a Stony Coral**

*Federica Scucchia^1^*†, Katrein Sauer^1^, Shah Fara^2^, Tali Mass^1^‡, Paul Zaslansky^2^‡**

^1^ Department of Marine Biology, Leon H. Charney School of Marine Sciences, University of Haifa; Haifa, Israel.

^2^ Department for Operative and Preventive Dentistry, Charité-Universitätsmedizin; Berlin, Germany.

*Corresponding authors. Email: federica.scucchia@studio.unibo.it, paul.zaslansky@charite.de

†Current address: Department of Biological Sciences, University of Rhode Island, Kingston, 02881, United States

‡These authors contributed equally

**Supporting Figures**


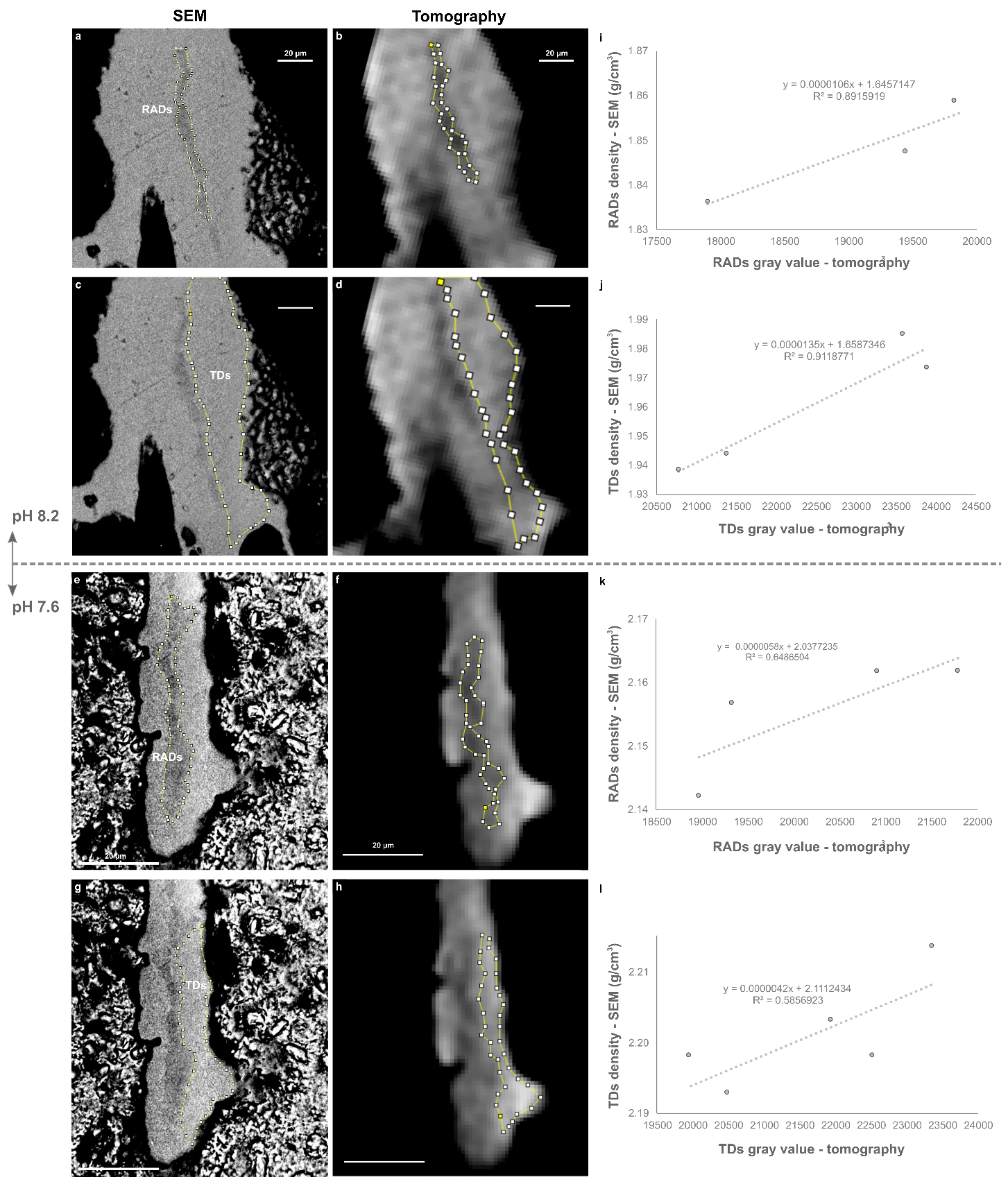


**Figure S1: Mineral density calibration.** (a-h) Mineral density of RADs and TDs was measured based on scanning electron microscope (SEM) images of normal pH (a, c) and low pH (e, g) corals and a known-density enamel sample. The same regions measured in the SEM images were measured in the X-ray absorption datasets of normal pH (b, d) and low pH (f, h) corals. (i-l) Density calibrations were performed by plotting the SEM-derived density values (g/cm³) against the tomography-derived gray values of each corresponding area. For both SEM and tomography regions, the mode was calculated and used for calibration.


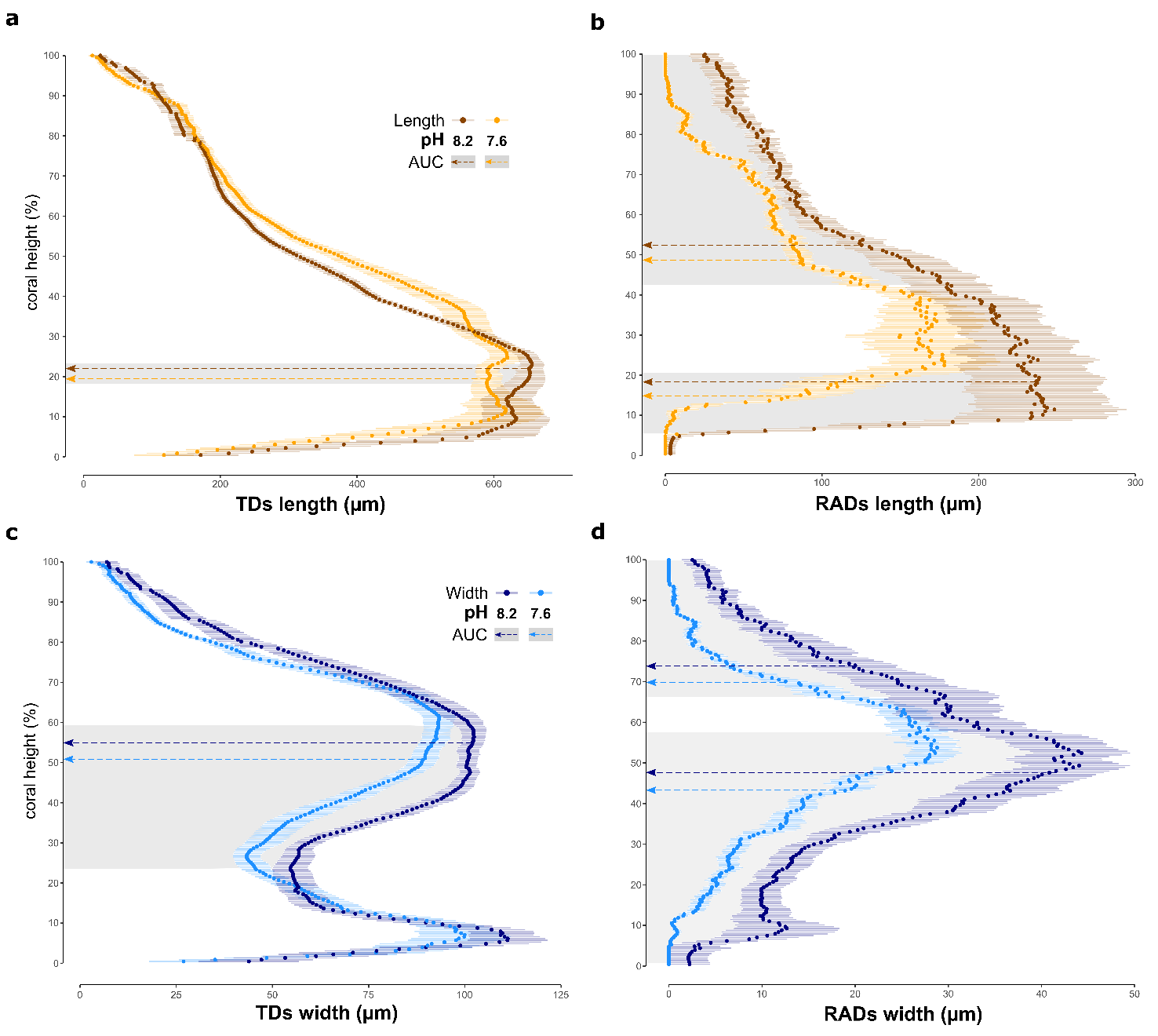


**Figure S2: Growth patterns of TDs and RADs under normal and OA conditions** (a, b) Cross-sectional length of TDs (a) and RADs (b) measured per all 6 primary septa of coral polyps, from the base to the top of the skeleton at the normal (8.2) and low (7.6) pH conditions. (c, d) Cross-sectional width of TDs (c) and RADs (d) at the normal and low pH conditions. Length and width were measured as the primary and secondary axis of the best-fitting ellipse circumscribing TDs and RADs in the tomographic slices. The shaded grey areas indicate the portions of the coral height where differences between corals at pH 8.2 (brown and blue arrows) and 7.6 (orange and light blue arrows) are statistically significant, as measured by comparing the area under the curve (AUC)(in these graphs it is the area enclosed within the y axis and the data points; unpaired t-test or Mann-Whitney test, p < 0.05; Table S1).


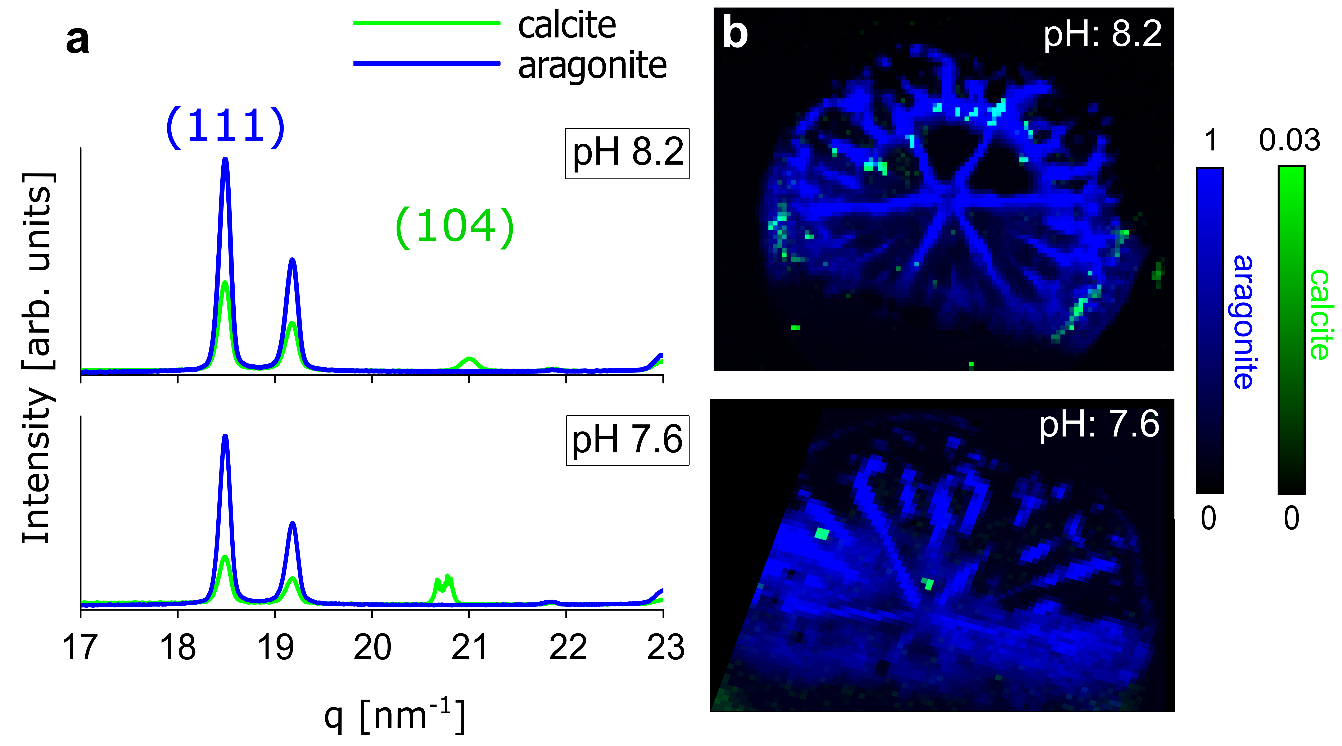


**Figure S3: Mineral phases within the coral skeleton**. (a) Typical peak profiles from XRD patterns illustrating aragonite (blue) and calcite (green) under normal (8.2, top) and OA (7.6, bottom) pH conditions (see Figure S9 for more details). (b) Two-dimensional XRD (2D-XRD) maps showing homogeneous distribution of aragonite across the entire sample, corresponding to the (111) peaks shown in panel (a), normalized to 1 for estimating calcite content.


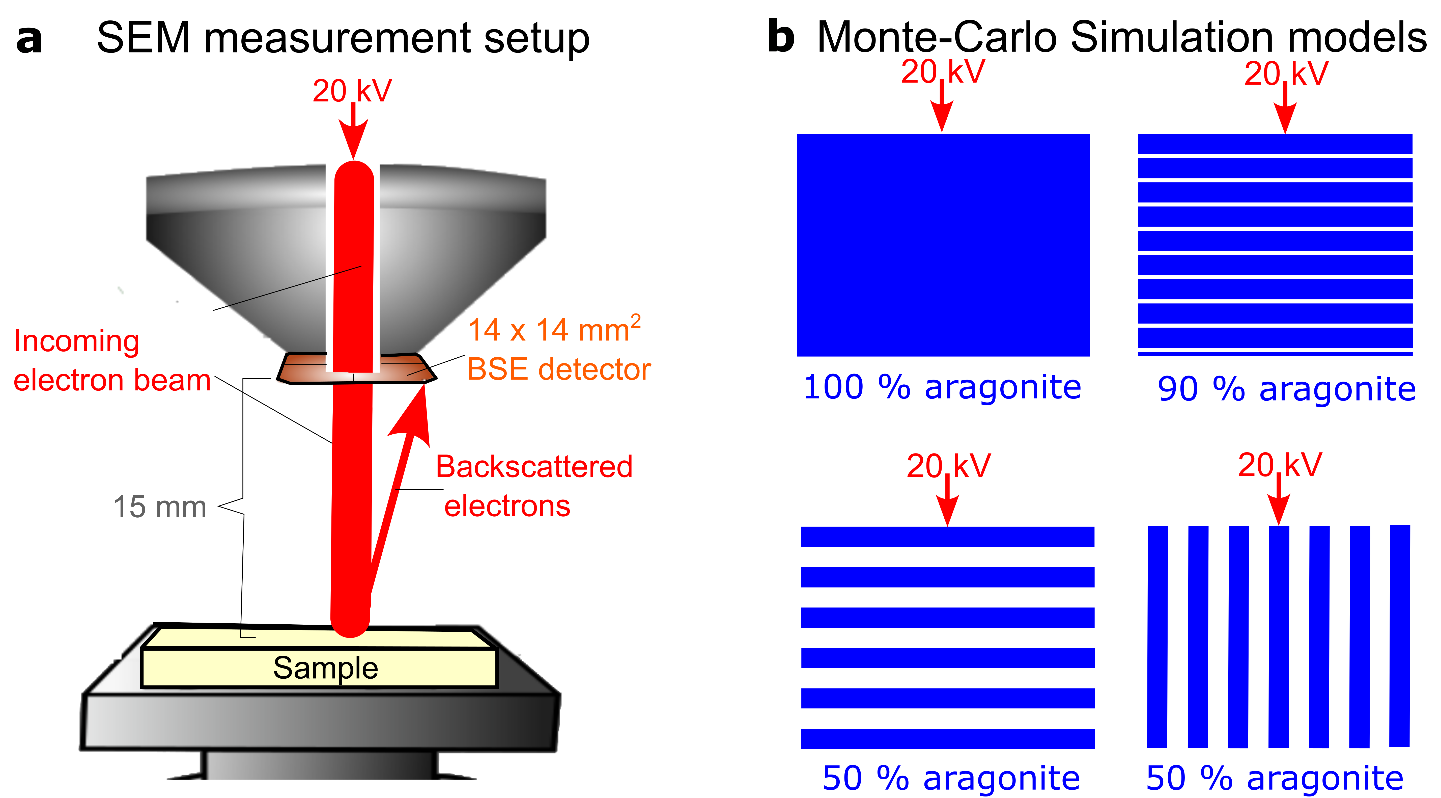


**Figure S4: Electron trajectories from electron microscopy experiments and Monte-Carlo simulations.** (a) Electron trajectories (red) as visualized in the electron microscope. The electron beam (red line) with an energy of 20 keV penetrates the sample (yellow), with electrons being back scattered (red arrow) and detected by the backscattered electron (BSE) detector (orange). (b) Simulation models of CaCO_3_ samples with varying elongated aragonite crystal compositions and orientations subjected to a 20 keV beam: (i) 100% pure aragonite, (ii) 90% aragonite arranged in layers perpendicular to the incoming electron beam, (iii) 50% aragonite oriented perpendicular to the incoming electron beam, and (iv) 50% aragonite oriented parallel to the incoming electron beam.


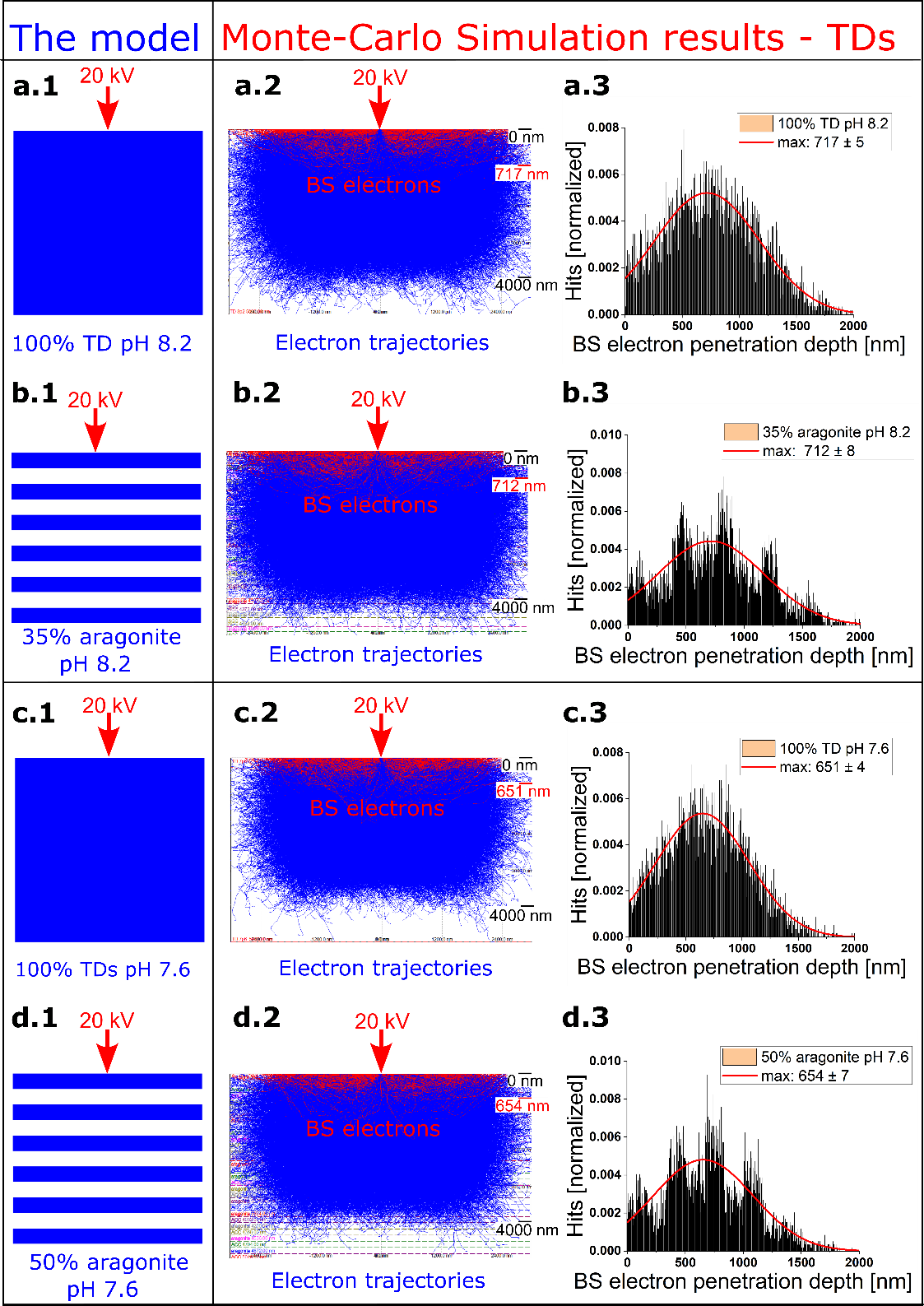


**Figure S5: Electron penetration depth in coral TDs corresponds to penetration depth in specific multi-layer aragonite-ACC configurations.** Monte-Carlo simulation models include: (a.1) 100 % coral TDs grown at pH 8.2, (b.1) 35 % aragonite, (c.1) 100 % coral TDs grown at pH 7.6, and (d.1) 50 % aragonite. For each model, (a.2.-d.2) depict simulated back scattered (BS) electron trajectories, while (a.3.-d.3) show material-specific BS electron penetration depth determined from the maxima of Gaussian distributions (red lines). To achieve matching penetration depth between multi-layer aragonite-ACC composites and TDs, 35 % of aragonite is required for pH 8.2-grown corals and 50 % aragonite for pH 7.6-grown corals.


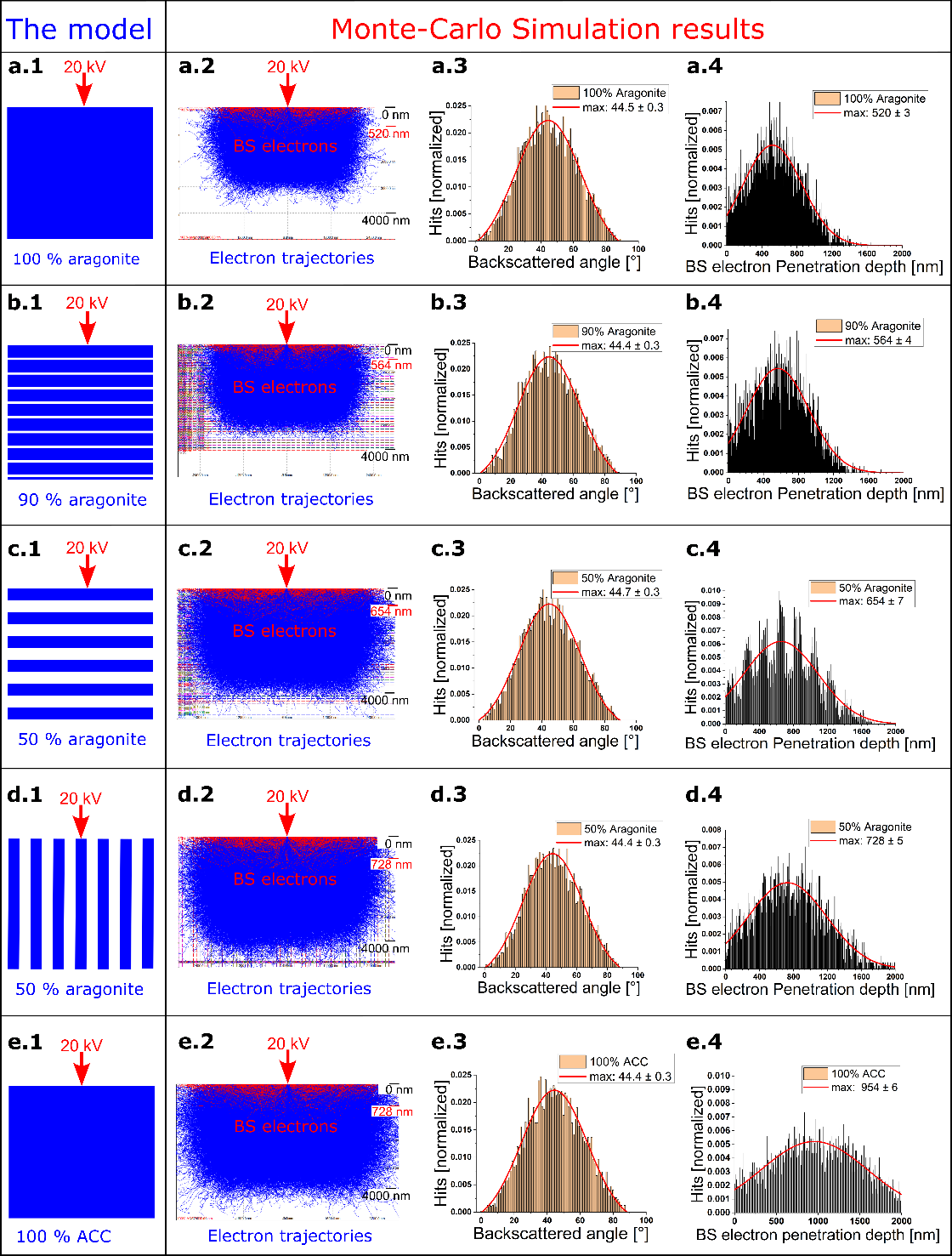


**Figure S6: Material properties of various models used in Monte Carlo simulations influence electron trajectories and penetration depth but not the angular distribution of back scattered (BS) electrons.** The Monte-Carlo models include: (a.1) 100 % aragonite, (b.1) 90 % aragonite layers and 10 % ACC layers, horizontally arranged, (c.1) 50 % aragonite layers and 50 % ACC layers, horizontally arranged, (d.1) 50 % aragonite layers and 50 % ACC layers, vertically arranged, and (e.1) 100 % ACC. For each model: (a.2.-e.2) displays simulated (BS) electron trajectories, (a.3.-e.3) present angular distributions of backscattered electrons determined from Gaussian maxima (red lines), which remain consistent across all models, and (a.4.-e.4) illustrate material-specific BS electron penetration depth derived from Gaussian maxima (red lines).


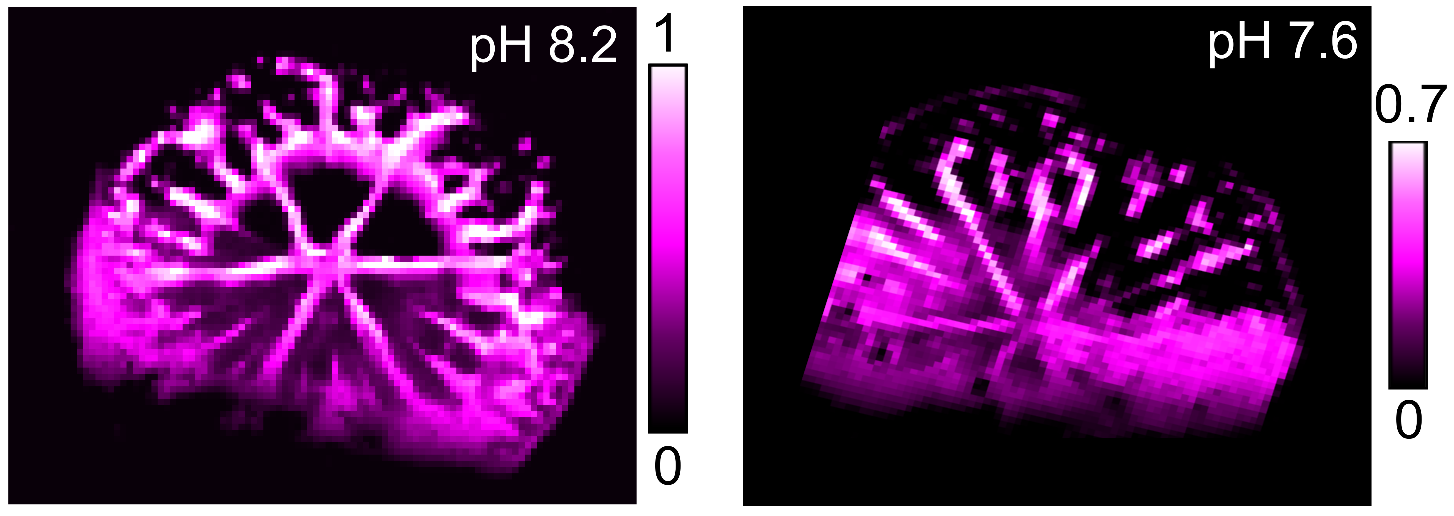


**Figure S7: Strontium distribution.** X-ray fluorescence (XRF) spectra depicting strontium distribution within corals growing at pH 8.2 and pH 7.6.


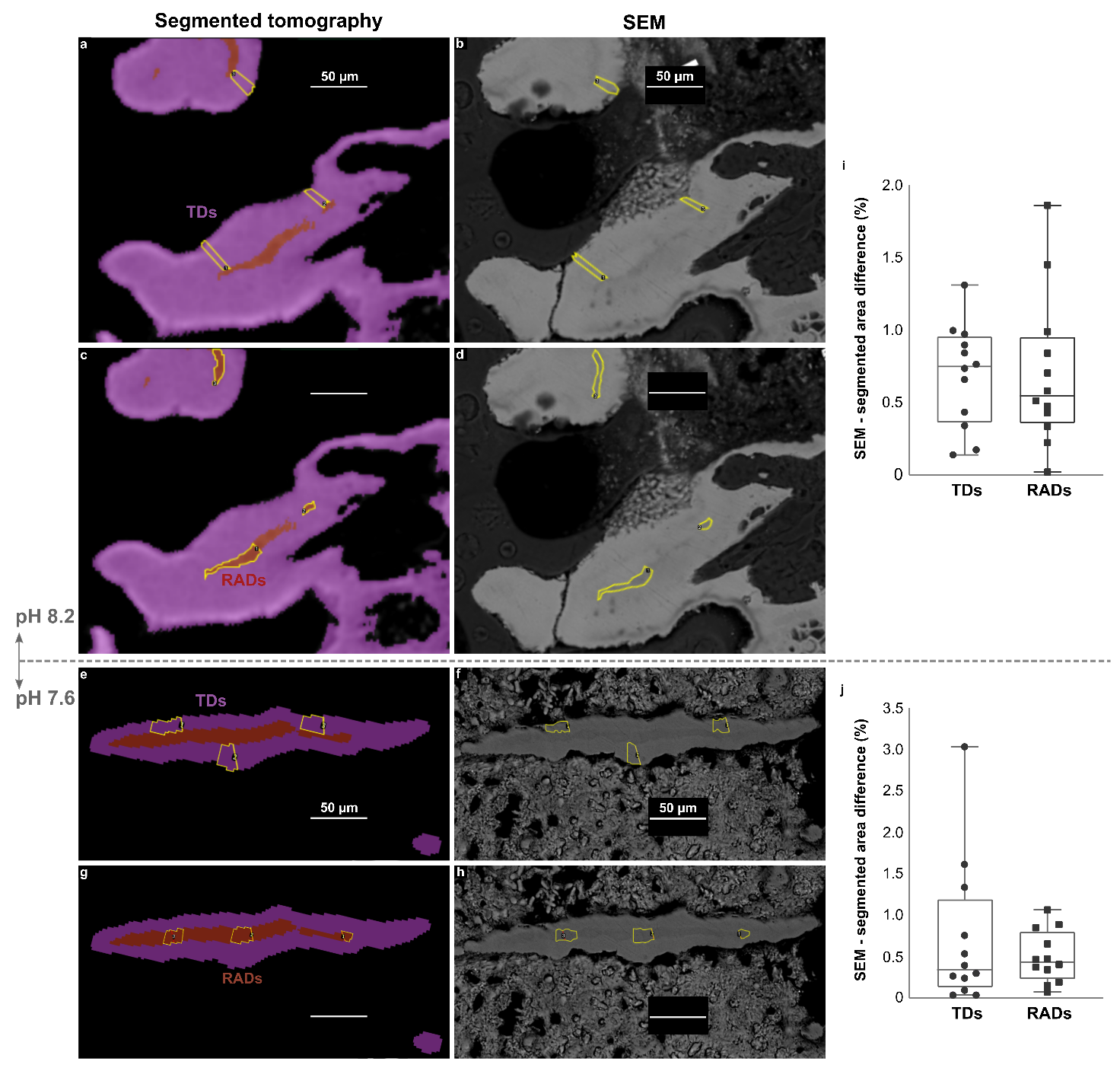


**Figure S8: Sensitivity of artificial intelligence-based segmentation.** (a-h) The same arbitrarily chosen regions within TDs and RADs were identified in tomographic cross-sectional slices segmented using artificial intelligence (AI)(panels a, c, e, g) and in the corresponding SEM images (b, d, f, h) for normal pH (a-d) and low pH (e-h) corals. (i, j) Box plots showing the difference (in percentage) between the SEM-based and the segmentation-based area measurements for TDs and RADs at the normal (i) and low (j) pH conditions. Solid horizontal bars correspond to median values, upper and lower hinges correspond to the first and third quartiles, whiskers extend to the lowest and highest values.


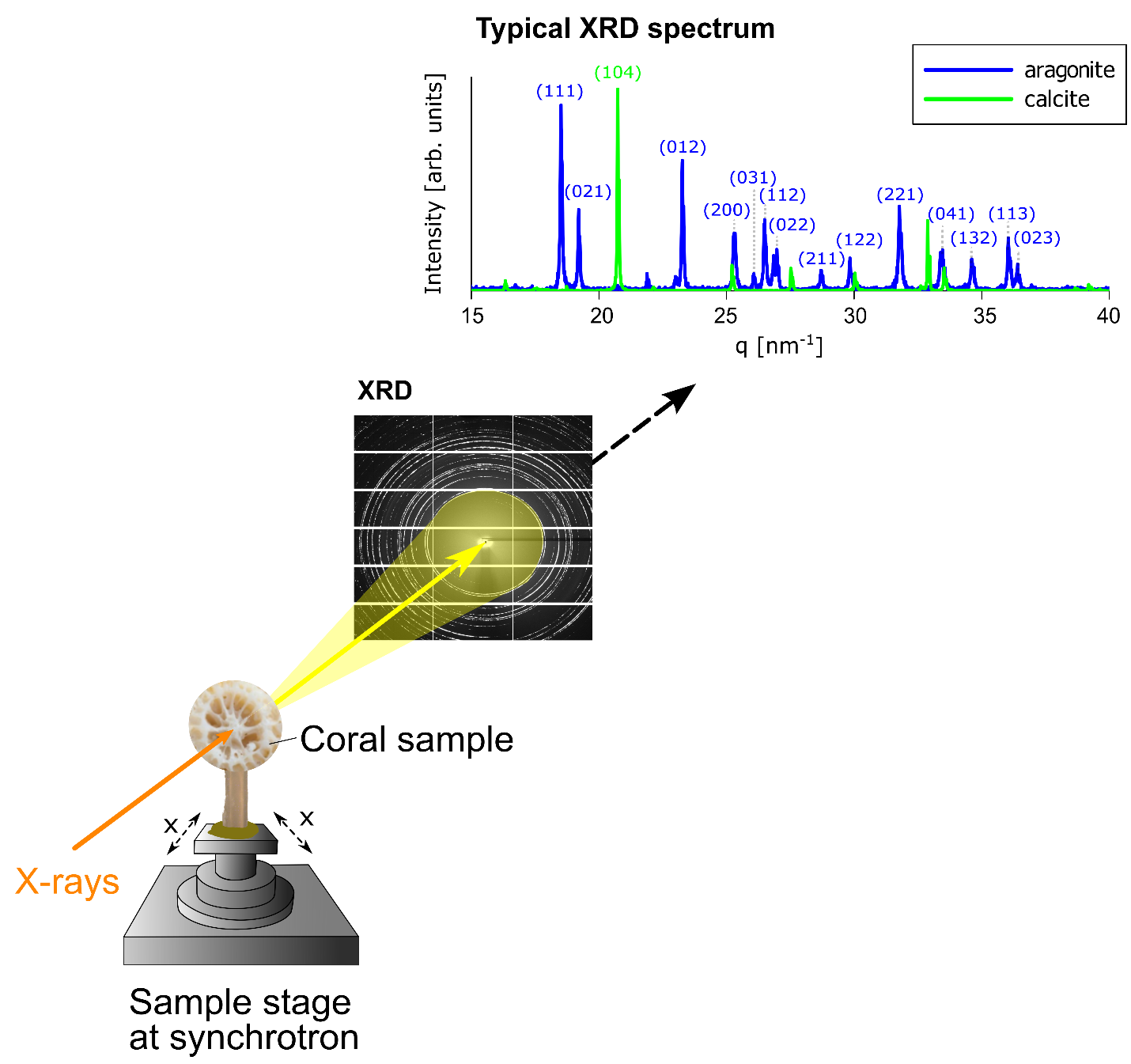


**Figure S9: XRD measurements over entire coral sample.** Coral samples were analyzed using XRD at the mySpot beamline of the BESSY II synchrotron light source (HZB- Helmholtz-Zentrum, Berlin, Germany), to generate mineral spectra.

**Table S1:** **Statistical tests results of TDs and RADs parameters measured at the normal and OA pH condition.** The number of replicates (n), t statistic value (t), degree of freedom (df) and P value (P) are reported for each variable examined (Unpaired t test or Mann-Whitney). Italic font style refers to cases analyzed with Mann-Whitney test. Region 1 and 2 refer to cases where there are multiple regions of the curves in Figure 4 and Figure S2 with a statistically significant difference among AUC, e.g. RADs area region 1 corresponds to the region of the curve in Figure 4b between ~5% and 57% coral height, and RADs area region 2 corresponds to the region of the curve in Figure 4b between ~64% and 100% coral height.

**
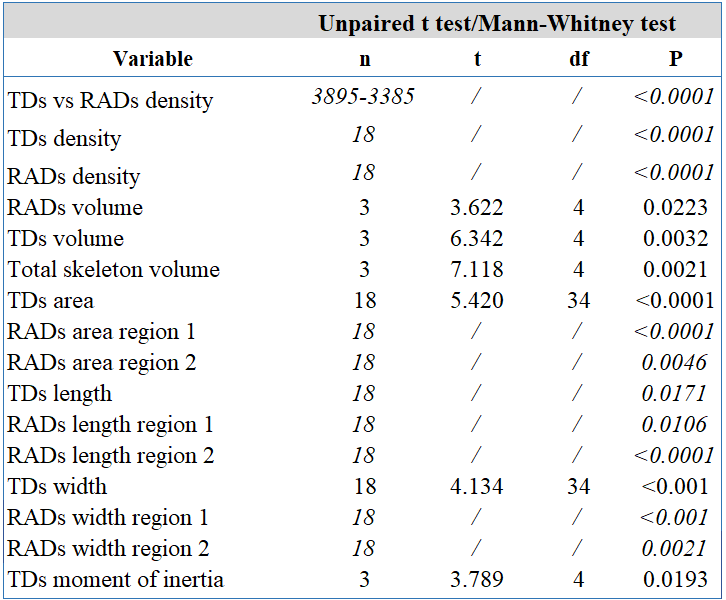
**

**Table S2:** **Seawater carbonate chemistry parameters across pH conditions**. Carbonate chemistry parameters were calculated per each experimental tank from measured values of temperature (T), salinity (S), pH (NBS) and total alkalinity (TA). For the entire duration of the experiment, measurements of temperature, salinity and pH were conducted three times a day, and measurements of total alkalinity were carried out once a day in triplicates for each tank. All values are shown as means ± SD. DIC, dissolved inorganic carbon; Ωar, aragonite saturation state. These measurements have been previously reported in^[24]^.


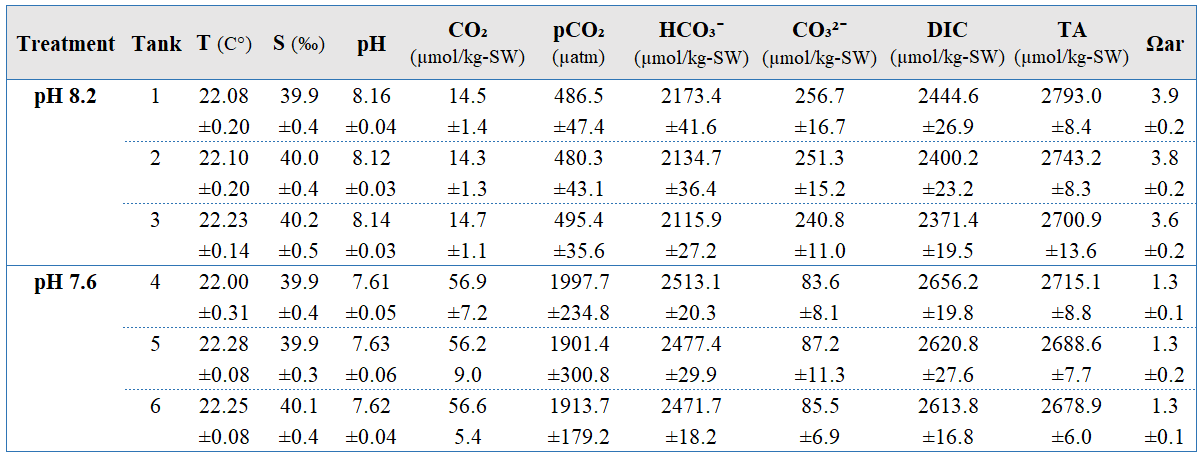

Supplement: Supplementary file 1 — Supporting Information [file ADVS-12-e08585-s002.docx]
